# Nitrogen Uptake Efficiency, Mediated by Fine Root Growth, Early Determines Temporal and Genotypic Variations in Nitrogen Use Efficiency of Winter Oilseed Rape

**DOI:** 10.3389/fpls.2021.641459

**Published:** 2021-05-13

**Authors:** Victor Vazquez-Carrasquer, Anne Laperche, Christine Bissuel-Bélaygue, Michaël Chelle, Céline Richard-Molard

**Affiliations:** ^1^Unité Mixte de Recherche ECOSYS, INRAE, AgroParisTech, Université Paris-Saclay, Thiverval-Grignon, France; ^2^IGEPP, INRAE, Institut Agro, Univ Rennes, Le Rheu, France

**Keywords:** *Brassica napus*, genetic variability, low-N inputs, Nitrogen Uptake Efficiency, Nitrogen Use Efficiency, roots, Specific Nitrogen Uptake

## Abstract

Maintaining seed yield under low N inputs is a major issue for breeding, which requires thoroughly exploiting the genetic diversity of processes related to Nitrogen Use Efficiency (NUE). However, dynamic analysis of processes underlying genotypic variations in NUE in response to N availability from sowing to harvest are scarce, particularly at the whole-plant scale. This study aimed to dynamically decipher the contributions of Nitrogen Uptake Efficiency (NUpE) and Nitrogen Utilization Efficiency (NUtE) to NUE and to identify traits underlying NUpE genetic variability throughout the growth cycle of rapeseed. Three experiments were conducted under field-like conditions to evaluate seven genotypes under two N conditions. We developed NUE_DM (ratio of total plant biomass to the amount of N available) as a new proxy of NUE at harvest, valid to discriminate genotypes from the end of inflorescence emergence, and N conditions as early as the beginning of stem elongation. During autumn growth, NUpE explained up to 100% of variations in NUE_DM, validating the major role of NUpE in NUE shaping. During this period, under low N conditions, up to 53% of the plant nitrogen was absorbed and NUpE genetic variability resulted not from differences in Specific N Uptake but in fine-root growth. NUtE mainly contributed to NUE_DM genotypic variation during the reproductive phase under high-N conditions, but NUpE contribution still accounted for 50–75% after flowering. Our study highlights for the first time NUpE and fine-root growth as important processes to optimize NUE, which opens new prospects for breeding.

## Introduction

Maintaining seed yield in a context of both increased climatic fluctuations and low nitrogen (N) inputs is a major issue for crop breeding and production. This is particularly relevant for winter oilseed rape *(Brassica napus L.)*, whose oil production represents ca. 15% of global vegetable oil production (FAOSTAT (Food and Agriculture Organization of the United Nations, Statistic Division), 2017), but has depended greatly on N fertilizers over the past several decades (Berry and Spink, 2006). N fertilization is the main expense in the economic cost of many crops (Rothstein, 2007; Kant et al., 2011), as well as a source of water pollution due to nitrate leaching (Di and Cameron, 2002) and air pollution due to N-derived greenhouse gas emissions (Sainju et al., 2012). Breeding oilseed rape varieties adapted to low N inputs could therefore ensure a more sustainable and competitive agriculture. This current challenge relies on increasing N Use Efficiency (NUE).

NUE results from the product of two interacting components, N Uptake Efficiency (NUpE), corresponding to the proportion of available N in the soil taken up by the crop, and N Utilization Efficiency (NUtE), corresponding to the conversion of this absorbed N into seed yield, i.e., grain yield per unit of N taken up (Moll et al., 1982). To date, most studies on oilseed rape NUE have focused on NUtE processes, assuming that the high N uptake capacities of oilseed rape (up to 100 kg N ha^−1^ before flowering) was not the main process that limited NUE (Rossato et al., 2001; He et al., 2017). Thus, ecophysiological processes related to N accumulation in the plant throughout the crop cycle, such as NUpE-related processes, i.e., Specific N Uptake (SNU), and root growth, remain relatively unexplored, particularly during the vegetative phase. However, Lemaire et al. (2008) showed that the amount of N absorbed before flowering has a major influence on the leaf area index, a key trait determining plant biomass production and the final number of seeds (Bissuel-Belaygue et al., 2011). The amount of N taken up before flowering could determine the yield potential (Colnenne et al., 2002; Svečnjak and Rengel, 2006a) which may depend on NUpE as well as on the architecture of the root system (Garnett et al., 2009; Ulas et al., 2012; He et al., 2017) and its genotypic variability (Wang et al., 2017). In addition, Richard-Molard et al. (2008) showed that the rate of N remobilization in response to N starvation in *Arabidopsis thaliana* is proportional to the amount of N previously accumulated, suggesting that remobilization efficiency during the reproductive phase may depend on NUpE during the vegetative phase. Thus, improving the processes underlying NUpE during the vegetative phase should be particularly relevant for optimizing NUE, especially under conditions of low N input. This improvement relies on the genetic diversity available within the germplasm of winter oilseed rape, as well as on targeting relevant ecophysiological traits to be examined. Crop breeders have largely overlooked root traits as selection criteria to improve NUE, due to the difficulty in measuring them under field conditions (Robinson, 2004). However, although few studies have considered the root compartment when characterizing genotypic variation in NUE in the field, some studies carried out on young plants in controlled conditions have highlighted the high genotypic variability in SNU and root architecture, traits that may influence NUpE (Laperche et al., 2006; Wang et al., 2017). No analysis of the genotypic variability of root architecture has yet been reported on mature plants, due to the persistence of phenotyping locks. Alternatively, a genotypic analysis of the fine-root growth, seen as a proxy of root architecture, could provide some clues of NUE determinism. Indeed, Wang et al. (2017) highlighted a strong correlation between root biomass and total root area on young plants of winter oilseed rape, and Louvieaux et al. (2020) evidenced a positive correlation between primary root length early measured in hydroponics and seed yield measured in the field.

To accelerate breeding programs, screening oilseed rape varieties at early stages of development is attractive but remains challenging. Finding traits that can be phenotyped early in the crop cycle and quickly on many genotypes, and be relevant for explaining differences observed at harvest is difficult, as evidenced by conflicting results in the literature. On the one hand, Balint and Rengel (2008) showed little consistency between the NUE of 12 oilseed rape varieties measured at the vegetative and maturity stages. On the other hand, Koscielny and Gulden (2012), as well as Louvieaux et al. (2020), found that seedling root length could be used as an early indicator of potential yield in winter oilseed rape and Wang et al. (2017) found QTL for root architecture traits that co-localized with QTL for NUE at the seedling stage.

However, an analysis of the dynamics of NUE components in response to N availability from sowing to harvest, highlighting the genetic variability in the underlying processes at the whole-plant scale, remains lacking. The objective of this study was to screen the main traits underlying the genotypic variation in NUE, including the fine-root compartment, with the aim to identify the main early contributors to NUE variations, particularly under low N supply. We proposed a three-step strategy. First, we investigated a new variable related to NUE as a tool to early screen genotypic variability in NUE. Second, we analyzed the relative contributions of NUpE and NUtE to NUE throughout the growth cycle under two contrasting N conditions. Third, we focused on the sub-processes underlying NUpE (i.e., SNU and fine root growth) by distinguishing fine roots from tap roots, considering that they do not have an equivalent role in N uptake, and we characterized their genetic diversity in a set of seven genotypes representing the germplasm of winter oilseed rape.

## Materials and Methods

### Plant Material

Seven lines of winter oilseed rape were investigated in three experiments under two contrasting N conditions (Table 1). Genotypes were chosen to represent genetic diversity in winter oilseed rape, both in terms of release date (1980–2004) and type (“++” vs. “00” types, with high vs. low glucosinolate and erucic acid contents, respectively) (Supplementary Table 1). They were selected from a panel of nearly 100 accessions, previously evaluated in the field (Bouchet et al., 2016), for their contrasting seed yield and NUE response to N inputs. Attention was paid to compare genotypes with similar growth-cycle durations and dates of flowering (no more than 8 days between the two extreme genotypes) to minimize confounding effects between phenology and NUE processes. The genotype AVISO was assessed as a control in all experiments.

**Table 1:** Overview of the experimental design: genotypes tested, substrates, nitrogen (N) conditions, and number of plant replicates per sampling date × genotype under each N condition in the three experiments.

**Experiment (code–site–year)**	**Genotype tested**	**Substrate**	**Nitrogen condition**	**Plant replicates per sampling date and genotype**	**Cumulative N supply**		**Substrate initial N amount**		**N available**		**Nitrogen nutrition index**			
					**g plant^**−1**^**	**kg ha^**−1**^**	**g plant^**−1**^**	**kg ha^**−1**^**	**g plant^**−1**^**	**kg ha^**−1**^**	**BBCH 16–18**	**BBCH 19**	**BBCH 30–32**	**BBCH 59**
**LR15** Le Rheu 2014-2015	AMBER ASTRID AVISO EXPRESS MOHICAN MONTEGO	Soil-Sand	Low-N High-N	5 5	0.22 1.47	25 165	0.18	21	0.40 1.65	46 186	0.82 0.90 ^*^	– – –	0.63 1.23 ^***^	– – –
**GR15** Grignon 2014-2015	AVISO	Attapulgite clay pebbles	Low-N High-N	6 6	0.26 1.56	29 175	0.25	28	0.51 1.81	57 203	– –	0.79 1.08 ^***^	0.72 0.96 ^***^	0.78 1.11 ^***^
**GR18** Grignon 2017-2018	AMBER AVISO EXPRESS MOHICAN OLESKI	Attapulgite clay pebbles	Low-N High-N	7 to 8 6	0.24 1.79	26 200	0.25	28	0.49 2.04	54 228	1.16 1.25 ^*^	0.76 0.84 ^*^	0.81 1.09 ^***^	0.67 1.03 ^***^

*In GR15 and LR15 experiments, each genotype was grown under the two N conditions, in contrast with GR18, where only AVISO was grown under the high-N condition. The mineral N available in pots for plant growth comes from that initially present in the substrate and that supplied by fertilization. The N Nutrition Index (NNI) was calculated on the genotype AVISO at four growing stages before flowering (BBCH 16–18, 19, 30–32, and 59). The significance codes (*

*** *P-value < 0.001*,

* *P-value < 0.05) refer to a comparison of NNI means carried out by pooling all genotype samples under the same N condition at the same sampling date in order to compare low-N and high-N conditions at four phenological stages (n = 30 for both N conditions in LR15, n = 6 for both N conditions in GR15 and n = 6 and 37 for high and low N-conditions respectively in GR18)*.

### Experimental Design

Three experiments (LR15, GR15, and GR18) were performed in two locations in France: LR15 was performed at Le Rheu (48°09′N, 1°76′W) during the 2014–2015 cropping season, while GR15 and GR18 were performed at Thiverval-Grignon (48°51′N, 1°58′E) during the 2014–2015 and 2017–2018 cropping seasons, respectively. Plants were grown on tubes 1 m high and 0.16 m diameter, with one plant per tube. Tubes were grouped into containers of 1 m^3^ (0.9 m wide × 1.2 m length × 1 m high), to reconstruct a canopy with a density of 35 plants m^−^^2^. Plants were grown outdoors, under conditions similar to those of field experiments for rain, radiation and wind. In the LR15 experiment, each tube was filled with 26.8 kg of a soil/sand mixture (60:40, v/v), yielding a bulk density of 1400 kg m^−3^. In the GR15 and GR18 experiments, each tube was filled with 10.2 kg of an attapulgite/clay pebble mixture (50:50, v/v), yielding a bulk density of 520 kg m^−3^. In the containers, the space between tubes was filled with a sand/soil mixture to keep all root sections at the same temperature. In addition, to avoid edge effects, two rows of plants were planted in the sand/soil mixture surrounding the tubes. This culture device provided access to the shoot and root systems (including fine roots) of each plant from sowing to maturity (Bissuel-Belaygue et al., 2015).

Six seeds of similar weight were sown in each tube from mid-September to mid-October. Seedlings were thinned twice during the first 2 weeks after emergence until only one medium-sized growing plant remained per tube. Pesticides were applied when necessary to control pests and diseases.

Experimental designs and sampling management are summarized in Tables 1, 2, respectively. In LR15, the experimental design consisted of a split-plot design with two N conditions as the main plot and six genotypes as sub-plots. In GR15, a single genotype (AVISO) was investigated under both N conditions, according to a complete randomized block design. In GR18, five genotypes were investigated under a single limiting N condition, except for AVISO, which was grown under both N conditions.

**Table 2:** Sampling management: phenological stages and climatic conditions at each sampling date of the three experiments.

**Experiment (code/site/year)**	**Sampling dates (day/month/year)**	**Days after sowing (DAS)**	**Phenological stages (BBCH scale)**	**Sum of growing degree-days (base 0**^****°****^**C)**		**Cumulative PAR (MJ m**^****−2****^**)**	
				**genotype mean**	**AVISO**	**genotype mean**	**AVISO**
**LR15** Le Rheu 2014-2015	26/01/15–30/01/15	94–98	18	802 ± 26	798 ± 26	174 ± 6	173 ± 6
	23/03/15–29/03/15	150–156	31	1148 ± 51	1153 ± 51	377 ± 25	378 ± 26
	11/05/15–27/05/15	199–215	68	1810 ± 204	1810 ± 10	772 ± 146	769 ± 9
	23/06/15–29/06/15	242–248	84 (Harvest)	2395 ± 117	2384 ± 0	1161 ± 80	1154 ± 0
			Δ Harvest-maturity	−355 ± 174	−366 ± 0	−69 ± 6	−77 ± 0
**GR15** Grignon 2014-2015	04/12/14–05/12/14	83–84	19		1041 ± 4		325 ± 5
	05/02/15–06/02/15	146–147	30		1321 ± 0		421 ± 3
	01/04/15–03/04/15	201–203	59		1653 ± 17		649 ± 6
	04/05/15–06/05/15	234–236	71		2052 ± 26		937 ± 19
	30/06/15–03/07/15	291–294	88 (Harvest)		2972 ± 78		1597 ± 41
			Δ Harvest-maturity		131 ± 0		69 ± 0
**GR18** Grignon 2017-2018	24/10/17–26/10/17	39–41	16	580 ± 27	565 ± 0	204 ± 6	200 ± 0
	12/12/17–14/12/17	88–90	19	905 ± 12	899 ± 0	302 ± 2	301 ± 0
	26/02/18–01/03/18	164–167	32	1289 ± 0	1289 ± 0	455 ± 13	449 ± 0
	10/04/18–13/04/18	207–210	59	1616 ± 26	1616 ± 7	655 ± 22	655 ± 5
	09/07/18	297	89 (Harvest)	3090 ± 0	3090 ± 0	1599 ± 0	1599 ± 0
			Δ Harvest-maturity	242 ± 109	262 ± 0	154 ± 0	154 ± 0

*Depending on the experiment, samples were taken at the beginning of rosette growth (BBCH 16–18), mid rosette development (BBCH 19), beginning of stem elongation after winter (BBCH 30–32), just before flower opening (BBCH 59), and end of the flowering period (BBCH 68–71). The final harvest (BBCH 84–89) was performed close to seed maturity and for all genotypes together, since the genotypes had similar phenology. The delta (Δ) between harvest (BBCH 84–89) and physiological seed maturity was calculated by assuming physiological seed maturity at BBCH 69 + 940 GDD (Jullien et al., 2011). The sum of growing degree-days (GDD) and the cumulative photosynthetically active radiation (PAR) from sowing are presented at each sampling date, for either the mean of all genotype samples under all N conditions in the same experiment (n = 60 in LR15 and n = 43 in GR18 at each sampling date) or for AVISO alone (n = 10 in LR15, n = 13 in GR18 and n = 12 in GR15 at each sampling date). The variation around each mean corresponds to the range of variation of these variables during the time required to harvest all plants for a given sampling date*.

### Climate Conditions

**Figure 1:**
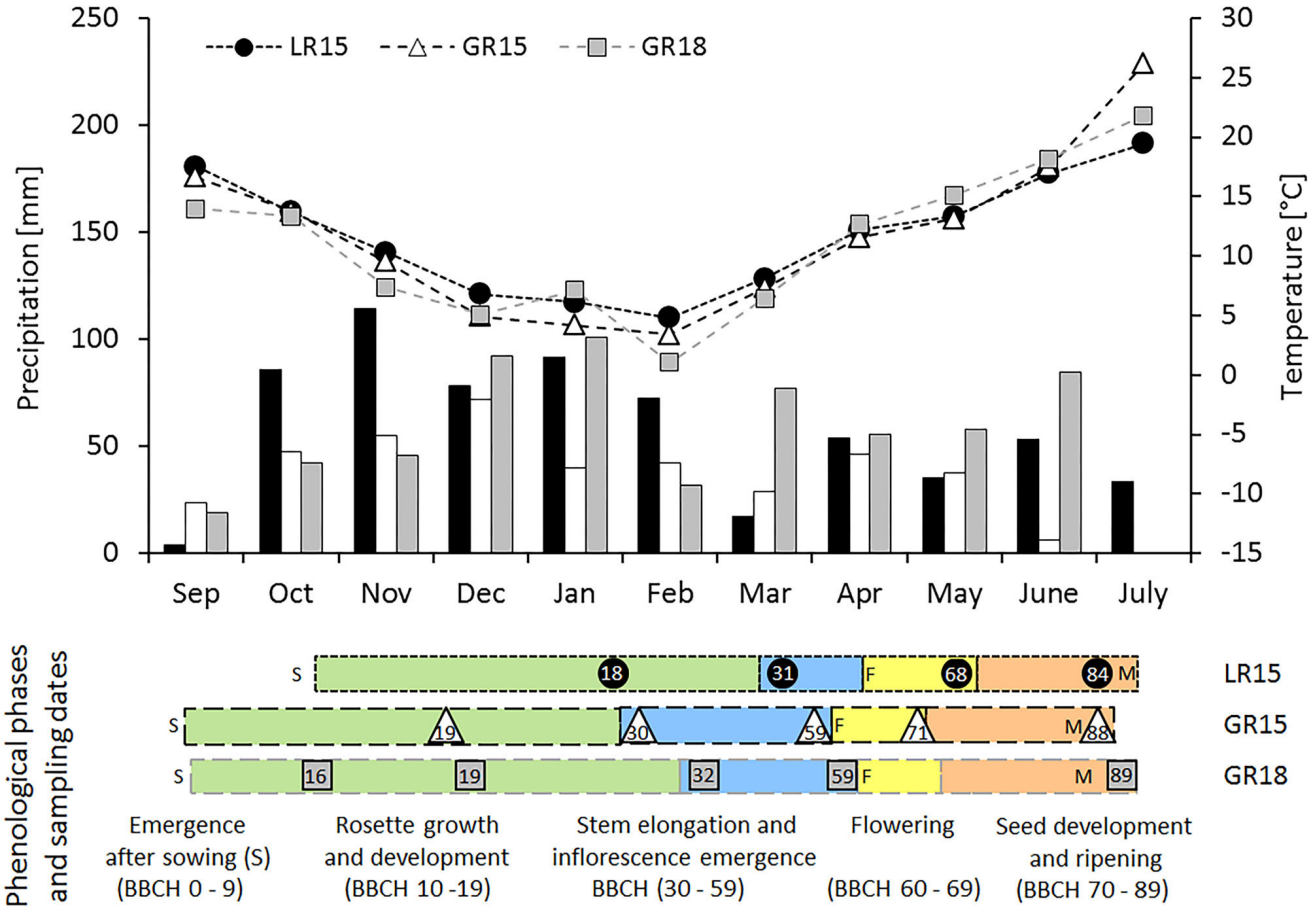
Total monthly precipitation (bars), mean monthly temperature (lines) and sampling dates and phenology of the AVISO genotype in the three experiments. Grayscale bars and symbols correspond to the three experimental sites × climate years (black circles, LR15; white triangles, GR15; gray squares, GR18). In the down panel, colors correspond to the four main phenological phases (green, rosette emergence, growth, and development; blue, stem elongation, and inflorescence emergence; yellow, flowering; orange, seed development, and ripening), sampling dates are indicated by symbols, whereas numbers depict phenological stages according to the BBCH scale (Lancashire et al., 1991). Letters correspond to experimental management (S, sowing) or phenological markers (F, beginning of flowering; M, seed maturity).

Daily mean air temperature (°C), precipitation (mm), photosynthetically active radiation (PAR, MJ m^−2^) and Penman evapotranspiration (mm) throughout the crop cycle were obtained from the INRA CLIMATIK^1^ platform. Growing degree-days (GDD) were summed from sowing using a base temperature of 0°C (Dresbøll et al., 2016). Because climate conditions differed among sites and years (Figure 1), the duration of the growing cycle varied among experiments: 242–248, 291–294, and 297 days for LR15, GR15, and GR18, respectively. For the genotype AVISO in LR15, GR15, and GR18, thermal time between sowing and seed maturity were 2384, 2972, and 3090 GDD, respectively, while cumulative PAR was 1154, 1597, and 1599 MJ m^−2^, respectively (Table 2).

Five phenological phases were defined according to the BBCH scale (Lancashire et al., 1991) to characterize the development of winter oilseed rape during the whole crop cycle: emergence (BBCH 0-9) rosette growth and development (BBCH 10–19), stem elongation and inflorescence emergence (BBCH 30–59), flowering (BBCH 60–69), and seed development and ripening (BBCH 70–89) (Figure 1). Beginning of flowering (BBCH 60) was assumed to be reached when 10 % of primary inflorescence flowers had opened, and seed maturity was assumed to be reached at BBCH 69 + 940 GDD (Jullien et al., 2011). The duration and climatic characteristics of the five phenological phases varied among experiments. LR15 had more precipitation and an overall deficit of cumulative PAR compared to GR15 or GR18 (Figure 1; Table 1). GR18 had the most GDD during stem elongation and flowering period. Thus, plants harvested at the same BBCH stage may have accumulated slightly different GDD and PAR values.

### Management of Hydric and Mineral Conditions

Each tube was watered to the soil's water holding capacity at the beginning and throughout the experiment with a modified Hoagland solution that provided no N [3 mM KCl, 1 mM MgSO_4_, 0.5 mM KH_2_PO_4_, 2.5 mM CaCl_2_, 27 μM Fe-EDDHA, 30 μM H_3_BO_3_, 10 μM MnSO_4_, 1 μM ZnSO_4_, 0.1 μM (NH_4_)_6_Mo_7_O_24_, 0.5 μM CuSO_4_, 0.5 μM CoCl_2_]. During experiments, cumulative precipitation (mm) and Penman evapotranspiration (mm) were used to estimate the soil water balance and manage Hoagland solution supplies to maintain soil moisture above 85% of field capacity, thus avoiding water stress and nutrient loss through leaching.

N was provided by a solution of KNO_3_ and Ca(NO_3_)_2_ (1:1 valence) mixed with the modified Hoagland solution and supplied every 200 GDD from emergence (BBCH 09) to harvest (BBCH 84–89), resulting in 13–14 applications during the growth cycle. The amounts of N applied per tube were calculated to generate two contrasting N conditions from emergence: low N, with a limiting cumulative N supply of 0.22–0.26 g per plant (equivalent to 25–29 kg N ha^−1^ in the field), and high N, with a non-limiting cumulative N supply of 1.47–1.79 g per plant (equivalent to 165–200 kg N ha^−1^) (Table 1). In addition to N applications, the mineral N initially present in the substrate was taken into account to quantify the mineral N available in the soil (Soil_QN). Homogeneous samples of substrate (50 g) were collected at three key stages common to all experiments (BBCH 16–18, BCH 30–32, and BBCH 59) at three depths (0–30, 30–60, and 60–90 cm) in several tubes to quantify water, ${\text{NO}}_{3}^{-}$ and ${\text{NH}}_{4}^{+}$ contents using the Kjeldahl (1883) method.

The N Nutrition Index (NNI) for C_3_ plants (Lemaire and Gastal, 1997) was used to quantify the plant N status generated by each N condition (Table 1). NNI was calculated as the ratio of N_obs_ to N_c_, where N_obs_ is the observed N content expressed as a percentage of shoot dry matter (W in t ha^−1^), and N_c_ is the critical N content determined for each given shoot biomass greater than or equal to 0.88 t ha^−1^ by the equation N_c_ = 4.8 W^−0.33^ established by Lemaire and Gastal (1997) for C3 crops. For shoot biomasses <0.88 t ha^−1^, Nc was set as a constant at 5.01%, which is the value given by the equation for *W* = 0.88 t ha^−1^. By comparing the observed N content to the critical N content of a crop at a given shoot biomass, NNI values below 1 indicate a N deficiency, while NNI values above one indicate a non-limiting N condition for plant growth.

### Sampling and Measurements

For each genotype and N condition, five to eight replicates were harvested at multiple phenological stages throughout the crop cycle (Figure 1; Table 2). In the LR15 experiment five replicates per treatment (i.e., genotype × N combination) were sampled at each sampling date (i.e., four times, including harvest). In the GR15 experiment, six replicates per treatment (i.e., N condition) were sampled at each sampling date (i.e., five times, including harvest). In the GR18 experiment, six replicates per genotype under the high N condition and seven to eight replicates per genotype under the low N condition were sampled at each sampling date (i.e., five times, including harvest) (Table 1; Supplementary Table 2).

The entire root system was carefully collected, paying special attention to recover all fine roots from the substrate. In addition, all senescing and dead leaves were counted and collected throughout the experiment. At each sampling date, harvested plants were divided into fractions: tap roots, fine roots, leaves (green, senescing, and dead), main stem, branch stems, and pods (including immature seeds or, when dehiscent, seeds, and pod walls). The dry matter (DM) of each plant fraction was weighed after lyophilization or oven drying at 70°C. Samples were then ground to a fine powder and analyzed for carbon (C) and N content according to the Dumas combustion method (Buckee, 1994), using an automated CN analyser (Vario MICRO Cube, Elementar France, Lyon, France). In the LR15 experiment, seed N content was estimated using near-infrared reflectance spectroscopy (MPA, Multi-Purpose FT-NIR Analyser, Bruker Optics, Ettlingen, Germany). Seed yield was calculated at the crop scale and expressed in t ha^−1^, considering a plant density of 35 plants m^−2^. The mean seed yield of AVISO was 6.03, 4.46, and 7.56 t ha^−1^ under the high-N condition and 2.02, 3.28, and 2.70 t ha^−1^ under the low-N condition in LR15, GR15, and GR18, respectively (Table 3), which matched with seed yields already reported in the field (Stahl et al., 2017, Corlouer et al., 2019).

**Table 3:** Effects of N condition and genotype on variation in the dry-matter-based N Use Efficiency (NUE_DM) and its components during the crop cycle in the three experiments.

**Sampling stage**		**GR18 (Grignon 2017–2018)**				**GR15 (Grignon 2014–2015)**				**LR15 (Le Rheu 2014–2015)**									
		**Low-N**		**%var**		**Low-N**	**High-N**	**%var**		**Low-N**		**High-N**		**Variance decomposition**					
**Trait**	**Unit**	**genotype mean**	**AVISO**	**G**		**AVISO**	**AVISO**	**N**		**genotype mean**	**AVISO**	**genotype mean**	**AVISO**	**G**		**N**		**GxN**	
**BBCH 16–*****18***																			
NUE_DM	g gN^−1^	1.39	1.28	57%	^***^	–	–	–		2.08	3.06	1.18	1.36	23%	^***^	31%	^***^	14%	^**^
NUpE	gN gN^−1^	0.07	0.07	53%	^***^	–	–	–		0.08	0.12	0.05	0.06	23%	^***^	30%	^***^	13%	^**^
NUtE	g gN^−1^	19.99	19.80	6%	ns	–	–	–		24.50	25.70	24.40	23.90	45%	^***^	<1%	ns	9%	ns
**BBCH 19**																			
NUE_DM	g gN^−1^	11.29	10.80	57%	^***^	19.57	12.23	55%	^**^	–	–	–	–	–		–		–	
NUpE	gN gN^−1^	0.39	0.37	62%	^***^	0.59	0.47	22%	ns	–	–	–	–	–		–		–	
NUtE	g gN^−1^	29.02	29.40	35%	^*^	33.04	25.63	89%	^***^	–	–	–	–	–		–		–	
**BBCH 30**–**32**																			
NUE_DM	g gN^−1^	23.62	24.30	15%	ns	28.45	12.73	86%	^***^	12.40	17.15	6.90	7.24	18%	^***^	48%	^***^	17%	^***^
NUpE	gN gN^−1^	0.68	0.71	6%	ns	0.68	0.42	65%	^**^	0.33	0.44	0.31	0.27	20%	^***^	33%	^*^	17%	^**^
NUtE	g gN^−1^	34.86	34.10	64%	^***^	39.04	30.07	68%	^***^	37.20	38.70	22.30	21.30	5%	^***^	87%	^***^	4%	^***^
**BBCH 59**																			
NUE_DM	g gN^−1^	36.02	42	68%	^***^	38.88	30.55	42%	^*^	–	–	–	–	–		–		–	
NUpE	gN gN^−1^	0.74	0.78	22%	ns	0.84	0.74	33%	ns	–	–	–	–	–		–		–	
NUtE	g gN^−1^	48.54	54.1	72%	^***^	46.39	40.76	40%	^*^	–	–	–	–	–		–		–	
**BBCH 68**–**71**																			
NUE_DM	g gN^−1^	–	–	–		70.83	33.74	99%	^***^	61.30	62.40	41.80	46.20	10%	ns	44%	^***^	7%	ns
NUpE	gN gN^−1^	–	–	–		0.83	0.74	60%	^**^	0.75	0.79	0.68	0.64	13%	ns	7%	^*^	9%	ns
NUtE	g gN^−1^	–	–	–		85.42	46.00	98%	^***^	82.80	82.50	62.00	72.20	32%	^***^	50%	^***^	4%	^*^
**BBCH 84**–**89**																			
NUE_DM	g gN^−1^	59.20	66.50	55%	^***^	72.03	27.96	98%	^***^	68.20	70.40	42.03	43.05	11%	^**^	62%	^***^	3%	ns
NUpE	gN gN^−1^	0.70	0.74	36%	^*^	0.75	0.51	94%	^***^	0.85	0.85	0.63	0.66	7%	ns	57%	^***^	5%	ns
NUtE	g gN^−1^	84.25	86.70	52%	^**^	95.84	54.75	97%	^***^	79.70	82.90	67.10	65.10	30%	^***^	43%	^***^	4%	ns
NUE_Seed	g gN^−1^	14.72	15.92	62%	^***^	18.33	7.02	98%	^***^	16.20	14.20	10.80	10.40	16%	^**^	42%	^***^	9%	^*^
Seed Yield	t ha^−1^	2.48	2.70	61%	^***^	3.28	4.46	76%	^**^	2.29	2.02	6.29	6.03	3%	^*^	85%	^***^	2%	ns

*Six key phenological stages were targeted: BBCH 16–18 (beginning of rosette growth), BBCH 19 (mid rosette development), BBCH 30–32 (beginning of stem elongation), BBCH 59 (just before flower opening), BBCH 68–71 (end of flowering), and BBCH 84–89 (seed maturity). For each stage, values of NUE_DM, NUpE, NUtE, and NUE_Seed represent the mean of all genotype samples cultivated under the same N condition in the same experiment (at each phenological stage, n = 30 in LR15 and n = 37 in GR18) and of AVISO alone (n = 5 in LR15, n = 7 in GR18, and n = 6 in GR15 at each phenological stage). The significance of genotype (G) (GR18 and LR15 only), nitrogen condition (N) (GR15 and LR 15 only), and genotype × N (GxN) interaction (LR15 only) effects was assessed for each experiment separately. Effects are expressed as a percentage of total variation (%var or, for LR15, variance decomposition). Significance codes*:

*** *P-value < 0.001*,

** *P-value < 0.01*,

* *P-value < 0.05*.

*NS, non-significant*.

### Variables Calculated

Depending on the variable considered, data were expressed either per plant fraction, per plant (all fractions), for shoot (aboveground fractions), or for roots (belowground fractions). Each variable was first calculated for each plant and then averaged either for each genotype under each N condition at each sampling date (*n* = 5 for LR15, *n* = 6 for GR15 and *n* = 6–8 for GR18) or by pooling all genotypes under the same N condition at each sampling date (*n* = 30 for LR15, *n* = 6 for GR15 and *n* = 6 and 37 under high and low N conditions respectively for GR18) (Supplementary Table 2).

At each sampling date, a DM-based NUE (NUE_DM, g gN^−1^) was calculated at the plant scale as the ratio of whole-plant DM (g plant^−1^, including tap and fine roots and senescing and dead leaves) to the quantity of soil mineral N available for plant growth (Soil_QN, gN plant^−1^), the latter being calculated by summing mineral N initially available from the soil and N applications (Equation 1):

(1) $$\begin{array}{l} NUE\text{\_}DM\;=\frac{Root\text{\_}DM\;+\;Shoot\text{\_}DM}{Soil\text{\_}QN} \end{array}$$

At harvest, NUE_DM was compared to NUE_Seed (g gN^−1^), calculated as the ratio of seed DM (g plant^−1^) to Soil_QN from sowing to harvest (gN plant^−1^).

NUpE (gN gN^−1^) was calculated as the ratio of the QN accumulated in the whole plant (Plant_QN) to Soil_QN (Equation 2):

(2) $$\begin{array}{l} NUpE\;=\frac{Root\text{\_}QN+\;Shoot\text{\_}QN}{Soil\text{\_}QN} \end{array}$$

NUtE (g gN^−1^) was calculated as the ratio of whole-plant DM to the QN accumulated in the whole plant (Equation 3):

(3) $$\begin{array}{l} NUtE\;=\frac{Root\text{\_}DM+\;Shoot\text{\_}DM}{Root\text{\_}QN\;+Shoot\text{\_}QN} \end{array}$$

Cumulative fine-root biomass was calculated from destructive measurements of fine-root DM. Two logistic functions were used to fit the dynamics of fine-root biomass accumulation under each N condition, one from emergence to winter and a second from winter to harvest, with the following logistic equation (Equation 4):

(4) $$\begin{array}{l} f\left(t\right)\;=\;c/\left(1+b\;\times \;e^{-at}\right) \end{array}$$

Parameters a, b and c were adjusted to minimize the sum of squares deviation, using the Generalized Reduced Gradient method for non-linear optimization (Lasdon et al., 1974). The integral of the fitted curve, representing cumulative fine-root biomass, was approximated using a Riemann's sum. It was used to calculate values of cumulative fine-root biomass at each sampling date.

SNU (gN g^−1^) was calculated as the ratio of the QN accumulated in the whole plant to cumulative fine-root biomass at each sampling date (Equation 4):

(5) $$\begin{array}{l} SNU\;=\frac{Root\text{\_}QN+\;Shoot\text{\_}QN}{Cumulative\;Fine\;Root\;DM} \end{array}$$

### Component-Contribution Analysis

The contribution of the components NUpE and NUtE to the variation in NUE_DM was calculated at each sampling date and analyzed under each N condition, as developed by Moll et al. (1982) and used for oilseed rape by Kessel et al. (2012) and Nyikako et al. (2014). Contribution analysis consists of linearizing the multiplicative relationship between NUE_DM, NUpE and NUtE by log-transforming it (Equation 6):

(6) $$\begin{array}{l} \log\;{\left(NUE\text{\_}DM\right)}_{i}=\;\log\;{\left(NUpE\right)}_{i}+\log\;{\left(NUtE\right)}_{i} \end{array}$$

The relative contribution of NUpE and NUtE (component traits) to the variation in NUE_DM (resultant trait) is then calculated according to Equations (7a,b), respectively:

(7a) $$\begin{array}{l} NUpE\;relative\;contribution=\\ \;\frac{\sum \log\;{\left(NUpE\right)}_{i}\;\times \;\log\;{\left(NUE\text{\_}DM\right)}_{i}}{\sum {\log\;\left(NUE\text{\_}DM\right)}_{i}^{\;2}} \end{array}$$

(7b) $$\begin{array}{l} NUtE\;relative\;contribution=\\ \;\frac{\sum \log\;{\left(NUtE\right)}_{i}\;\times \;\log\;{\left(NUE\text{\_}DM\right)}_{i}}{\sum {\log\;\left(NUE\text{\_}DM\right)}_{i}^{\;2}} \end{array}$$

### Statistical Analysis

Statistical analyses were performed and plots were generated using R software v. 3.4.2 (R Core Team, 2018). Pearson's correlation coefficients (r) were calculated from the means of all genotypes and Holm's correction was applied for the evaluation of correlation significance. Parameters of non-linear models (i.e., logistic curves and exponentials) were adjusted using the nls function (non-linear least squares) (Bates and Watts, 1988; Bates and Chambers, 1992). Linear regression models were fitted with the lm (linear models) function. Type II analyses of variance (ANOVA) were performed using the “car” package of R, and Tukey's *post-hoc* procedure was used to compare means. ANOVA assumptions were tested using the Shapiro-Wilk and Levene's tests. Hotelling (1931) T-squared distribution test was used to test the multiple parameters of the non-linear models. Statistical significance was estimated at α = 5%.

## Results

### Relating NUE_Seed to NUE_DM at Seed Maturity and at Earlier Stages

**Figure 2:**
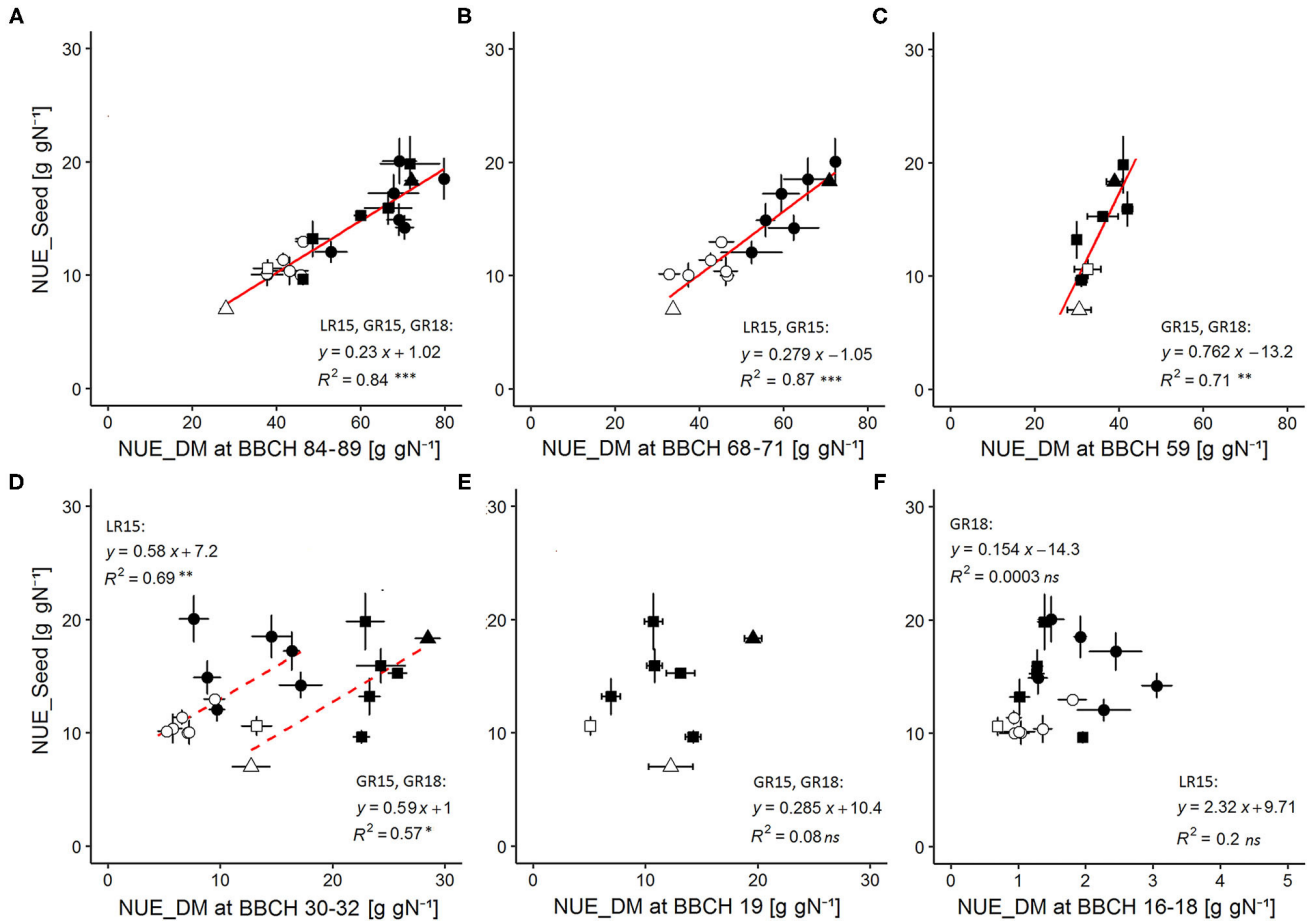
Relationships between NUE_Seed measured at seed maturity (BBCH 84-89) and NUE_DM throughout the crop cycle. NUE_DM was measured at **(A)** seed maturity (BBCH 84-89), **(B)** end of flowering (BBCH 68-71), **(C)** just before flower opening (BBCH 59), **(D)** beginning of stem elongation after winter (BBCH 30-32), **(E)** mid rosette development (BBCH 19), and **(F)** early rosette development (BBCH 16-18). Regressions were performed by pooling data for all sites × years × N conditions when no significant differences on relationships parameters were found by separately comparing site, year or N condition effects. Solid lines indicate significant regressions (*P*-value < 0.05) validated both for each N condition separately as well as by pooling N conditions. Dashed lines indicate significant regression (*P*-value < 0.05) valid only when grouping N conditions. Open symbols correspond to the high-N condition, while filled symbols correspond to the low-N condition, with circles for LR15, triangles for GR15, and squares for GR18. Error bars depict standard errors of the mean of each genotype with *n* = 5 and 6 at each sampling date and for each N condition in LR15 and GR15, respectively, and *n* = 6 and 7–8 at each sampling date for high and low N-conditions respectively in GR18.

We aimed at validating NUE_DM as a new variable reliably reflecting NUE_Seed variations at seed maturity. NUE_Seed values of the AVISO genotype ranged from 7.02 to 10.40 g gN^−1^ under the high-N condition and from 14.20 to 18.33 g gN^−1^ under the low-N condition (Table 3). At seed maturity (BBCH 84–89), NUE_DM of AVISO ranged from 27.96 to 43.05 g gN^−1^ under the high-N condition and from 66.50 to 72.03 g gN^−1^ under the low-N condition (Table 3). Interestingly, by pooling data from all sites, years, genotypes, and N conditions, we identified a strong and unique linear relationship between NUE_Seed and NUE_DM calculated at harvest (*R*^2^ = 0.84; *P*-value = 1 × 10^−8^) (Figure 2A), highlighting that NUE_DM at seed maturity was closely related to NUE_Seed, regardless of genotype, climatic condition, or N condition.

We investigated this relationship throughout the crop cycle to determine how early NUE_DM became a good proxy for NUE_Seed. NUE_DM increased continuously during the crop cycle, due to the continuous increase in the total plant biomass (since dead leaves were included) relative to the quantity of N available in the soil (Table 3). In addition, NUE_DM was always 1.3–2.6-fold higher under the low-N condition than the high-N condition (*P*-value < 0.001). We tested the relationship between NUE_Seed (calculated at seed maturity) and NUE_DM calculated at five earlier phenological stages: end of flowering (BBCH 68–71), just before flower opening (BBCH 59), beginning of stem elongation (BBCH 30–32), and mid- and early rosette growth (BBCH 19 and BBCH 16–18, respectively) (Figures 2B–F). Regardless of N condition and genotype, NUE_Seed had a strong relationship with NUE_DM at BBCH 68–71 (*R*^2^ = 0.87; *P*-value = 1.4 × 10^−6^) and BBCH 59 (*R*^2^ = 0.71; *P*-value = 0.009) (Figures 2B,C). Moreover, we were able to evidence that the relationship was common to both experimental sites at BBCH 68–71, and to both experimental years at BBCH 59. At BBCH 30–32, site-specific relationships were observed, due to differences in intercept but with the same slope (Figure 2D). The relationships observed at BBCH 30–32 were mainly driven by N conditions, as they became not significant when considering genotypes in a single N condition. At BBCH 19 and BBCH 16–18, the relationships became non-significant (Figures 2E,F). All the significant relationships were shown to be also valid in each separate N condition and experiment (Supplementary Table 3). Thus, NUE_DM measured as early as BBCH 59 could be used as a robust proxy trait to represent NUE_Seed of genotypes at seed maturity in all N conditions. At BBCH 30–32, the proxy is still valid to discriminate N conditions but not accurate enough to discriminate genotypes.

The relationships between NUE_DM at seed maturity and that earlier in the growth cycle were similar (*R*^2^ = 0.88, *P*-value = 7 × 10^−7^ and *R*^2^ = 0.75, *P*-value = 5 × 10^−3^ at BBCH 68–71 and BBCH 59, respectively; *R*^2^ = 0.71, *P*-value = 1 × 10^−3^ and *R*^2^ = 0.73, *P*-value = 7 × 10^−3^ at BBCH 30–32 for LR15 and GR15 + GR18, respectively) (data not shown), indicating that NUE_DM may also be a relevant variable for dynamically clarifying NUE shaping in various genotypes as early as BBCH 59 and in response to N supply as early as BBCH 30–32.

### Dynamic Contribution of NUpE and NUtE to NUE_DM

**Figure 3:**
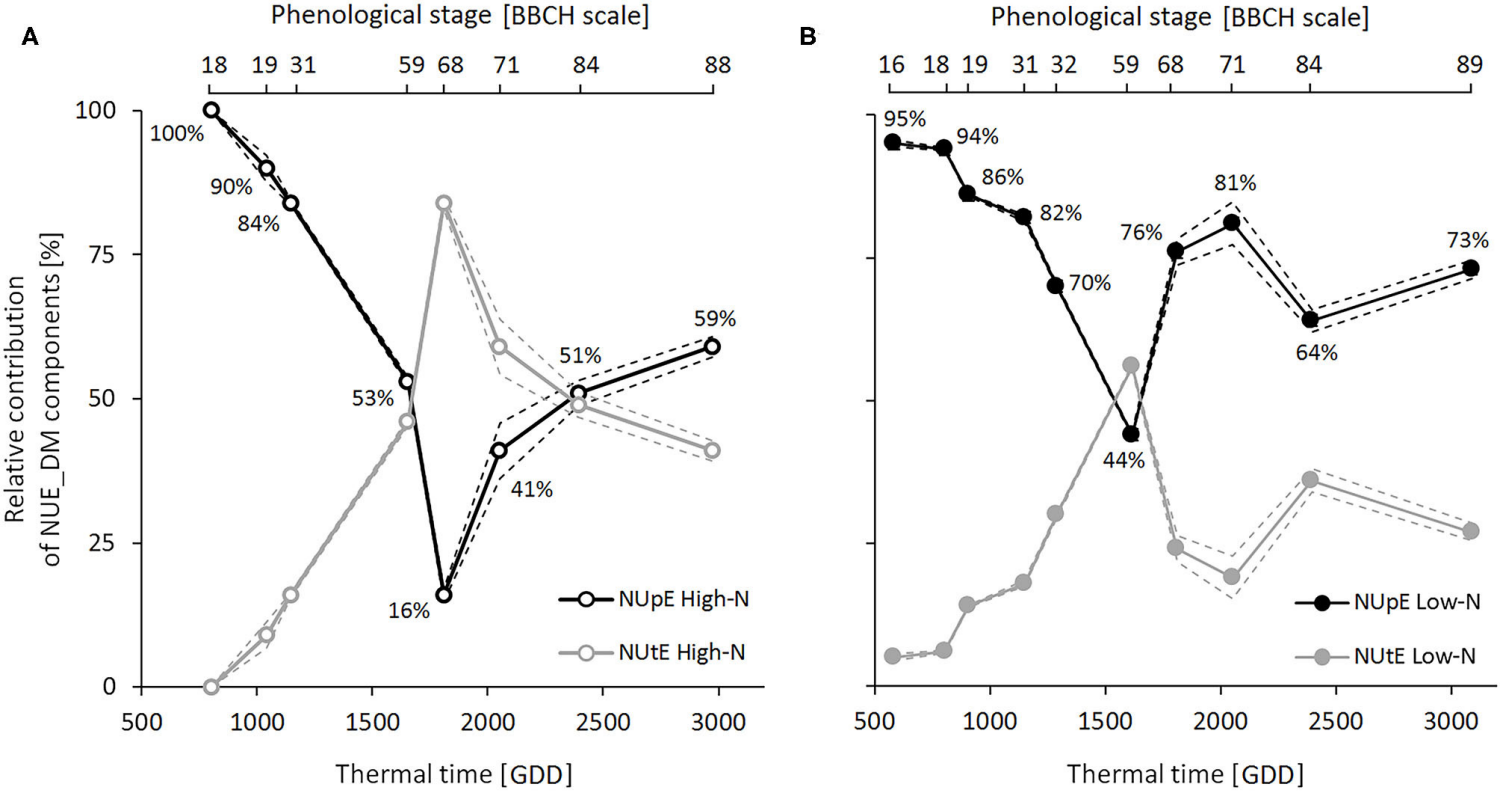
Contribution dynamics of N Uptake Efficiency (NUpE) and N Utilization Efficiency (NUtE) to variations in dry-matter-based N Use Efficiency (NUE_DM) throughout the crop cycle under high-N **(A)** and low-N **(B)** conditions. Relative contributions were calculated by pooling samples of all genotypes from LR15, GR15, and GR18 experiments (*n* = 6–37 for the low N-condition and *n* = 6–30 for the high-N condition at each sampling date). Black and gray symbols correspond to NUpE and NUtE, respectively. Dashed lines depict 95% confidence intervals. The percentage values correspond to the NUpE contributions to NUE_DM.

Dynamic analysis of the two NUE components at six sampling dates during the crop cycle indicated that NUpE and NUtE, like NUE_DM, were lower under the high N condition than the low N condition (Table 3). Three contrasting phases emerged from the dynamic analysis of relative contributions of NUpE and NUtE to NUE_DM throughout the crop cycle (Figure 3). During the vegetative phase (BBCH 16-18–59), the contribution of the NUpE was first predominant (95–100%) and then decreased steadily up to flowering, but still accounted for 44–53%, regardless of the N condition. During the flowering period (BBCH 60–69), contrasted patterns distinguished according to N conditions. In the high N condition, the relative contributions of NUpE and NUtE inverted, with NUtE becoming the main NUE_DM component (84%) and the contribution of NUpE decreasing sharply to 16%. In contrast, in the low N condition, the contribution of NUpE increased strongly again to reach 76%. Finally, during seed development and ripening (BBCH 70–89), the contribution of NUpE leveled off at 64–81% under the low N condition, and 41–59% under the high N condition, with a trend to increase during seed ripening under both N conditions, indicating that NUpE still played a significant contribution during this phase, especially when N supply was limiting.

### Genotypic Variation in NUE_DM and Its Components

A high and significant genotypic variability in NUE_DM was identified using the LR15 and GR18 datasets, since the genotype effect explained up to 68% of the overall variation in NUE_DM, depending on the site and climate year (Table 3). In the GR18 experiment, the genotype effect on NUE_DM variance remained strong and significant throughout the crop cycle, except at BBCH 30–32 where it was not significant. In contrast, in the LR15 experiment, the genotype effect decreased progressively during the crop cycle, leveling off around 10% during the reproductive phase. In both experiments, the highest and most significant genotypic effect was observed during the vegetative phase, suggesting that the NUE_DM genotypic variability was determined from the beginning of the vegetative phase.

**Figure 4:**
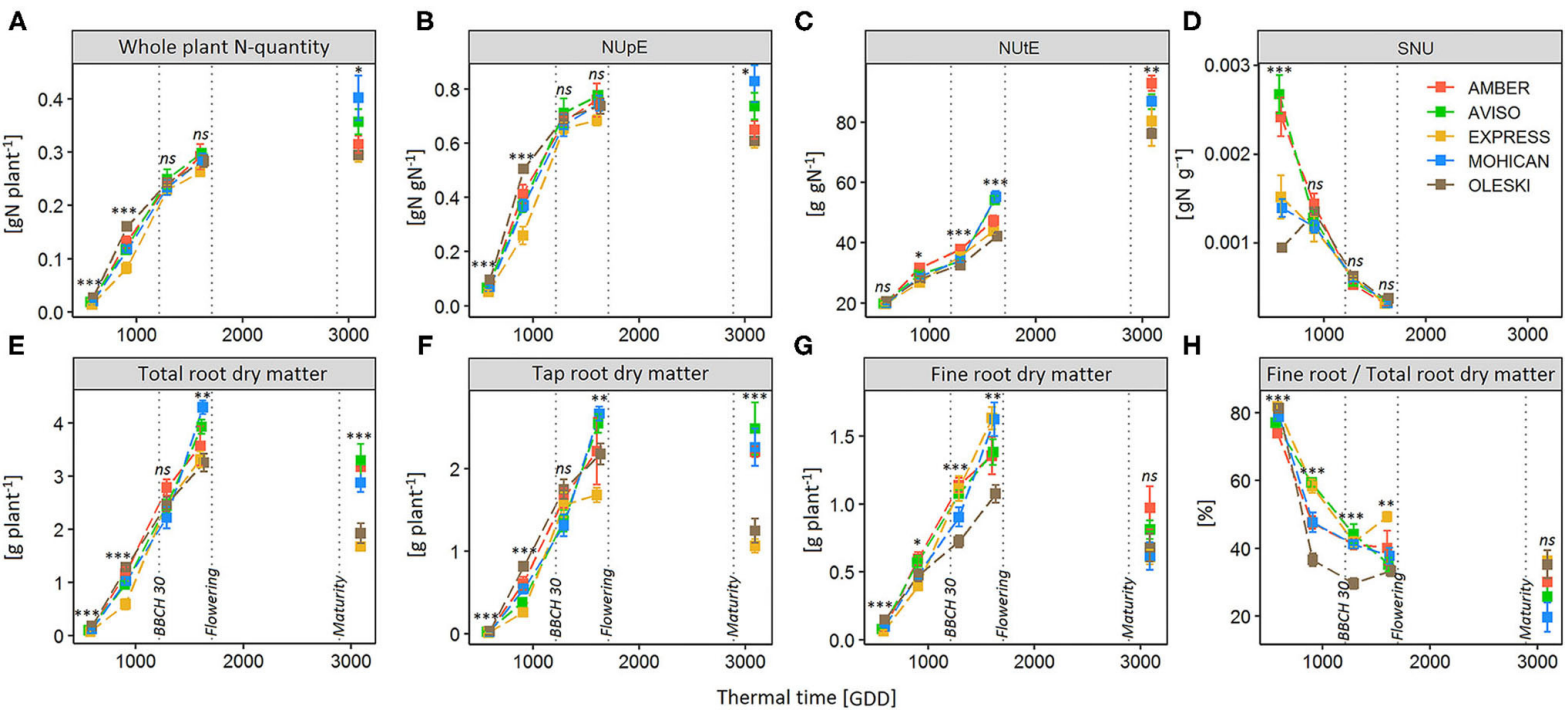
Dynamics of N accumulation in the whole plant **(A)**, N Uptake Efficiency (NUpE) **(B)**, N Utilization efficiency **(C)**, Specific Nitrogen Uptake (SNU) **(D)**, total-root **(E)**, tap-root **(F)** and fine-root **(G)** dry matter, and fine-to total root ratio **(H)** during the crop cycle for five genotypes (AMBER, AVISO, EXPRESS, MOHICAN, OLESKI) grown under the low-N condition (GR18 experiment). Each genotype is represented by a different color. Vertical dotted lines depict the end of winter (BBCH 30), the beginning of flowering (BBCH 60), and the seed maturity (BBCH 84). Error bars indicate standard errors of the mean of each genotype, with *n* = 7–8 at each sampling date. Significance codes: ^***^P-value < 0.001, ^**^P-value < 0.01, ^*^P-value < 0.05, ns, non-significant.

As for NUE_DM, in both experiments, the genotypic effect in NUpE variations was the highest and the most significant during the beginning of the vegetative phase. Indeed, genotype effects on NUpE were significant only up to the beginning of stem elongation (BBCH 31), except a genotype effect observed at harvest in GR18 (Table 3). In the GR18 experiment, NUpE increased up to the end of the vegetative phase (BBCH 59), and then leveled off (AVISO and MOHICAN) or slightly decreased (AMBER, EXPRESS, and OLESKI) during the reproductive phase (Figure 4). At BBCH 59, AVISO and MOHICAN had the highest NUpE (0.78 and 0.75 gN gN^−1^, respectively), whereas EXPRESS had the lowest (0.68 gN gN^−1^) (Supplementary Table 4). Even if the differences were not significant, this tendency may explain the significant differences observed in NUpE at seed maturity.

On the other hand, NUtE significantly differed between genotypes from BBCH 18 onwards (Table 3) and slight differences between AMBER and EXPRESS appeared at BBCH 19 (Supplementary Table 4; Figure 4). From BBCH 32 to harvest, NUtE increased for all genotypes and three groups of genotypes can be distinguished: AVISO and MOHICAN which had high NUtE values at BBCH 59 and harvest, EXPRESS and OLESKI which had the lowest values of NUtE both at BBCH 59 and harvest, and finally AMBER which had one of the lowest values of NUtE at BBCH 59 (not significantly different from EXPRESS and OLESKI) but the highest NUtE value at harvest (not significantly different from AVISO and MOHICAN). In any case, the percentage of NUtE variance explained by the genotype effect increased from BBCH 59 onwards, far exceeding that of NUpE.

Significant genotype x nitrogen interactions (GxN) were also observed on the NUE_DM and NUpE variances during vegetative growth (Table 3). From BBCH 68–71, they became non-significant. For NUtE, GxN were significant at BBCH 30–32 and BBCH 68–71. Those GxN effects accounted for almost the same percentage of variance as the genotype effect at BBCH 30–32 on the LR15 experiment, with 4 vs. 5% of variance percentage for the GxN vs. G effect on NUtE, respectively; 17 vs. 20% on NUpE; and 17 vs. 18% on NUE_DM.

We also analyzed how variations in NUpE and NUtE observed among genotypes reflected variations observed among genotypes in NUE_DM, using correlation analyses based on mean values per genotype (Table 4). During the beginning of the vegetative phase, strong and significant correlations were found between NUE_DM and NUpE under both N conditions (r ≥ 0.97 for BBCH 16–18 to BBCH 19). The correlations were still strong and significant at BBCH 31 (LR15 experiment), but no longer at BBCH 32 (GR18 experiment). From BBCH 59 onwards, the correlation between NUpE and NUE_DM was no longer significant. Correlations between NUtE and NUE_DM were non-significant up to BBCH 30–32, except a significant correlation observed at BBCH 31 under the low N-condition in LR15. From BBCH 59 onwards, higher significant correlations were found between NUE_DM and NUtE in GR18 and in LR15 under the high N condition, but not under the low N-condition. No significant correlation was observed between NUE_DM and NUtE at harvest. Thus, the genetic variability highlighted mainly during autumn growth for NUpE could be exploited to early tune NUE_DM.

**Table 4:** Correlation analysis between dry-matter-based N use efficiency (NUE_DM) and N uptake Efficiency (NUpE) or N Utilization Efficiency (NUtE) under low-N and high-N conditions during the crop cycle.

	**LR15**				**LR15**				**GR18**			
	**High-N**				**Low-N**				**Low-N**			
	**NUpE**		**NUtE**		**NUpE**		**NUtE**		**NUpE**		**NUtE**	
BBCH 16–18	0.98	^***^	–	ns	0.99	^***^	–	ns	1.00	^***^	–	ns
BBCH 19	–	*na*	–	*na*	–	*na*	–	*na*	0.97	^*^	–	ns
BBCH 30–32	0.97	^**^	–	ns	0.99	^***^	0.93	^*^	–	ns	–	ns
BBCH 59	–	*na*	–	*na*	–	*na*	–	*na*	–	ns	0.97	^**^
BBCH 68–71	–	ns	0.95	^**^	–	ns	–	ns	–	*na*	–	*na*
BBCH 84–89	–	ns	–	ns	–	ns	–	ns	–	ns	–	ns

*Six key phenological stages were targeted: BBCH 16–18 (early rosette development), BBCH 19 (mid rosette development), BBCH 30–32 (beginning of stem elongation), BBCH 59 (just before flower opening), BBCH 68–71 (end of flowering), and seed maturity (BBCH 84–89). For each sampling date, analyses were performed on the mean values of each of the winter oilseed rape genotypes grown under low-N conditions at Grignon (GR18) (n = 7–8 at each sampling date) and under low-N and high-N conditions at Le Rheu (LR15) (n = 5 at each sampling date). The table shows Pearson's correlation coefficients (r) and significance of each regression, with*

*** *P-value < 0.001*,

** *P-value < 0.01*,

* *P-value < 0.05*.

*Holm's correction was applied for the evaluation of correlation significance. Dashes correspond to non-significant (ns) or not available (na) correlations*.

### Deciphering Genotypic Variation in NUpE-Related Processes Under Low N Conditions

The above results highlighted NUpE as the main contributor to NUE_DM shaping, especially under low N conditions and as an important driver of its genotype variability during the autumn growth. This raises the question of identifying the key processes underlying this trait. NUpE depends on the quantity of N accumulated in the plant, itself driven by two root processes: ability to absorb N per unit of cumulative fine-root biomass (SNU) and ability to maximize exchange area with the soil through root system development, that we approximated by fine-root growth. The study was focused on low-N conditions and carried out on the GR18 dataset that characterizes several genotypes with sufficient sampling frequency to ensure the establishment of reliable cumulative fine-root growth dynamics. However, the same trends were also observed in the LR15 data under low N-conditions, produced at a lower sampling frequency and on different genotypes (Supplementary Table 5).

The Plant_QN dynamics of each genotype under low N conditions (Figure 4) showed that most N was taken up during the vegetative phase, since genotypes had accumulated 73–93% of their final Plant_QN by the beginning of flowering, and even mainly during the autumn growth (up to BBCH 32), which already represents 58–82% of the total nitrogen absorbed, depending on the genotype (Supplementary Table 4). However, all genotypes maintained N uptake during the reproductive phase, although the amount and percentage of the N taken up from flowering to seed maturity varied greatly among genotypes (from 4 to 29% for OLESKI and MOHICAN, respectively; Supplementary Table 4).

**Figure 5:**
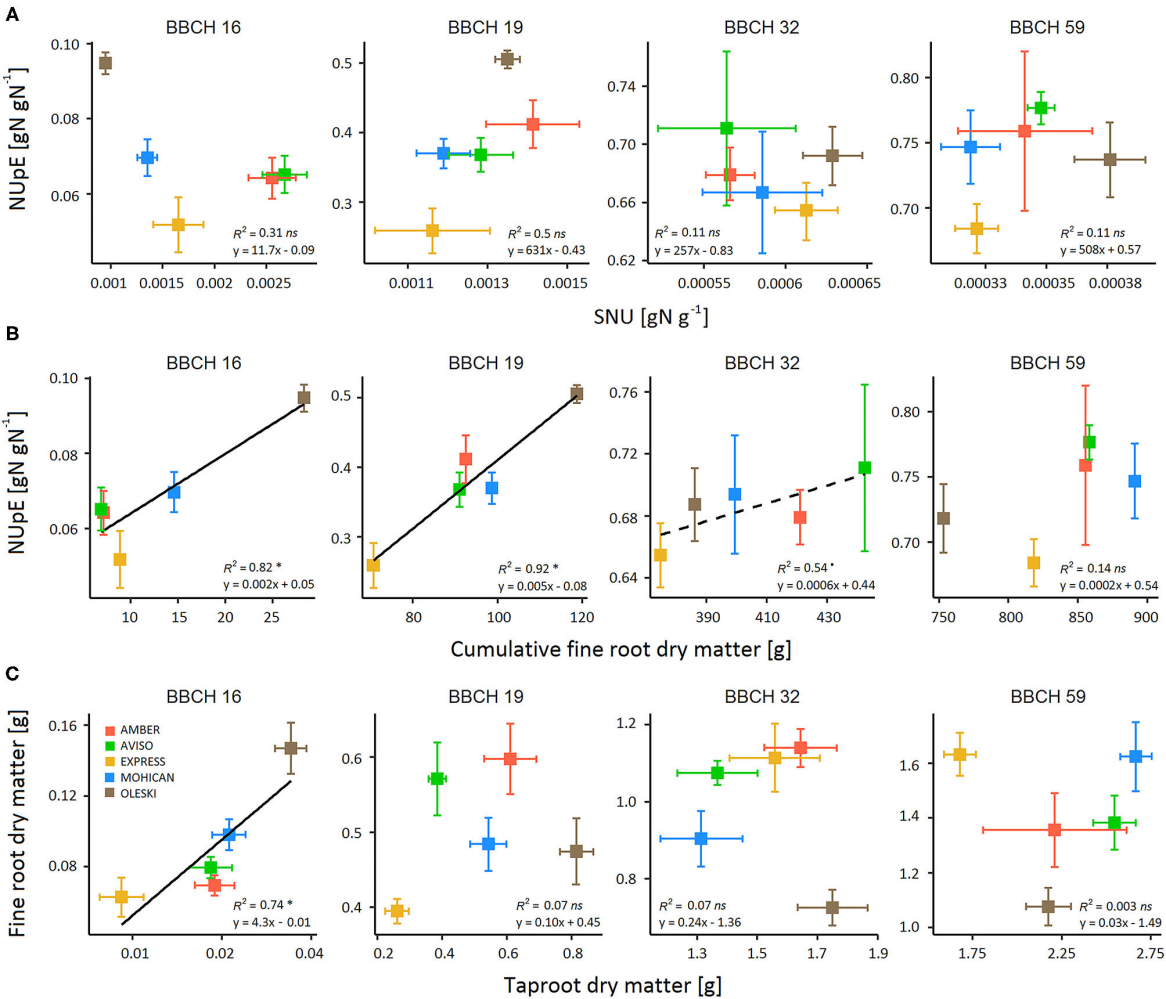
Relationship between **(A)** NUpE and specific N uptake (SNU), **(B)** Nitrogen Uptake Efficiency (NUpE) and cumulative fine-root dry matter, and **(C)** fine root and tap root dry matter during the vegetative growth (BBCH 16–59) for five winter oilseed rape genotypes under low-N conditions (GR18 experiment). Colored squares indicate the mean value per genotype. Error bars indicate standard errors of the mean of each genotype with *n* = 7–8 at each sampling date. Significant regressions at *P*-value < 0.05 are indicated by a solid line and (^*^) symbol, while significant regressions at *P*-value < 0.1 are indicated by a dashed line and (.) symbol.

Genotypes did not differ significantly on SNU, except at BBCH 16–18 (Figure 4; Table 3). In addition, NUpE was never significantly correlated to SNU (Figure 5A), suggesting that SNU was not the main driver of genotypic nor temporal variability in NUpE.

In contrast, significant positive relationships were found between NUpE and cumulative fine-root biomass (Figure 5B) from the beginning of rosette development (BBCH 16, *R*^2^ = 0.82) to the beginning of stem elongation (BBCH 32, *R*^2^ = 0.54). Moreover, fine-root biomass had high genotypic variability during the vegetative phase, but not at seed maturity (Figure 4; Supplementary Table 4). Cumulative fine-root biomass could then be considered as a relevant trait for characterizing NUpE variation between genotypes. In addition, a unique relationship was shown between Plant_QN and cumulative fine-root biomass, regardless of genotype (Supplementary Figure 1), suggesting that genotype differences in Plant_QN raised more from differences in cumulative fine root dry matter than in SNU.

As fine roots are tedious to phenotype in the field or on mature plants, we investigated if total- or tap-root biomasses could be used as proxies of fine root biomass. Like fine roots, total- and tap-root biomasses had high genotypic variability during the vegetative phase (Figure 4; Supplementary Table 4). No significant correlation was found between fine-root and total root biomasses, nor between fine-root and tap-root biomasses, except at the beginning of rosette development (BBCH 16), when tuberization was low (Figure 5C). But they exhibited independent genotypic variations, suggesting that genotypes differed in the partitioning of root biomass. Thus, neither taproot nor total root biomass could be used as a proxy of fine-root biomass.

## Discussion

Our objective was to analyze dynamics of NUE and its components and decipher the processes underlying their genotypic variability, which would merit consideration in breeding programs for low N-input systems. Our study was based on NUE_DM, which we propose as a new variable to monitor NUE shaping accurately throughout the crop cycle. Comparing to the alternative variables already developed (Craswell and Godwin, 1984; Raun and Johnson, 1999; Svečnjak and Rengel, 2006a), NUE_DM offers the advantage of assessing NUE at each phenological stage and of considering all plant compartments, including fallen leaves, tap roots, and fine roots. NUE studies usually neglect these organs because they are difficult or tedious to harvest, especially in the field or at the plant scale. However, our results indicated that the contribution of fine roots and fallen leaves to whole-plant biomass, and thus to NUE, is far from negligible. Under the low-N condition, fine roots represented up to 32% of total biomass during the vegetative phase and fallen leaves up to 26% at harvest. Regarding N, fine roots and fallen leaves represented up to 21 and 26.5%, respectively, of the N taken up from sowing to harvest. These data are consistent with the results of Malagoli et al. (2005a), who reported a loss of 12% of plant N in fallen leaves. Some other studies assessing NUE include tap roots and sometimes fine roots, but not fully for each individual plant (Ulas et al., 2012; Hohmann et al., 2016; Yuan et al., 2016, Dresbøll et al., 2016) or at each phenological stage, mainly targeting either the seedling or reproductive stages (Thomas et al., 2016; Wang et al., 2017; Louvieaux et al., 2020). To our knowledge, our study is the first to describe the NUE dynamics of winter oilseed rape so completely and accurately, by considering entire individual plants grown under canopy conditions at key phenological stages from sowing to seed maturity.

As the first main finding, our study highlights the early determinism of NUE. Indeed, NUE_DM was a proxy trait of NUE_Seed valid as early as the end of inflorescence emergence (BBCH 59) to discriminate genotypes, and the beginning of stem elongation (BBCH 32) to discriminate N conditions. Correlations were validated for the range of climatic conditions observed in our three experiments, highlighting the robustness of the proxy, even if it should be assessed in more extreme environmental conditions. Thus, for the purposes of genotypic scoring, it may be sufficient to phenotype NUE just before flowering. Some correlations have also been reported in the literature between NUE_Seed and total biomass (Stahl et al., 2019), but only at flowering and not before. Thus, our results highlight the role of the vegetative phase in the determinism of NUE.

Our study is the first to dynamically quantify the relative contribution of NUpE to NUE throughout the crop cycle. Our second main finding is the identification of NUpE as the main contributor to NUE during the whole vegetative phase and particularly during autumn growth. Indeed, up to the beginning stem elongation (BBCH 31), NUpE contributed more than 80% to NUE_DM variations, and NUpE genotypic variations strongly correlated to those of NUE_DM. NUE_DM would thus rely mainly on N uptake processes during this period. Accordingly, Cramer (1993) showed that 35% of the total amount of N taken up by the time of harvest had already been taken up by the end of autumn growth. In our study, these values raised up to 53% of the total amount of N under low N-conditions. This discrepancy could be due to differences in the N balance sheet, which included all plant compartments in our study; and differences in N supply dynamics, which led to higher N availability during the autumn growth, compared to field conditions. Thus, autumn growth was the period during which NUpE strongly determined variations in NUE_DM and also the period during which most of the N was absorbed.

Interestingly, we also showed that NUpE continued to contribute strongly to NUE after flowering in our experiments, with a relative contribution of 59% under the high-N condition and 73% under the low-N conditions at harvest. Using the same contribution analysis, Kessel et al. (2012) on rapeseed and Rakotoson et al. (2017) on rice obtained similar ranges of values at harvest, also with a higher relative contribution of NUpE vs. NUtE especially under low N conditions. Accordingly, we showed that N uptake continued after flowering, contrary to observations of Malagoli et al. (2005b) but consistent with those of Berry et al. (2010), Schulte auf'm Erley et al. (2011), and Ulas et al. (2012). The proportion of N taken up during the reproductive phase varied according to genotype, but reached up to 29%, which highlights contrasting genotypic behavior in the management of N uptake dynamics, consistent with contrasted NUpE and NUtE values. Thus, NUpE continued to play a significant role during seed development and ripening, especially when N supply was limited.

Even if the contribution of NUtE remained overall low under the low-N condition, NUtE was predominant to explain NUE_DM variations from flowering under the high-N condition. The contributions of NUtE to NUE_DM variations ranged from 41 to 89% and significant correlations between NUE_DM and NUtE genotypic variations were observed as soon as BBCH 59 under low N conditions and at BBCH 68–71 under high N conditions. Kessel et al. (2012) also pointed out that the genotype variations in NUE at seed maturity were mainly due to differences in NUtE under high N conditions. These results suggested that N utilization processes balanced N uptake processes during the reproductive phase under plethoric N conditions, i.e., when N is largely stored into the plant, which is consistent with previous studies (Girondé et al., 2015a,b) that have highlighted the importance of genotypic variability in the remobilization processes to improve NUE.

The high N uptake capacities and the poor N remobilization capacities from senescing leaves of rapeseed during vegetative phase (Dejoux et al., 2000; Malagoli et al., 2005a; Girondé et al., 2015b) led to the widely held assumption that NUpE is not a lever for increasing NUE (Svečnjak and Rengel, 2006a; Avice and Etienne, 2014). Our results contradict this assumption, suggesting that N uptake could become a relevant lever for increasing NUE in low N-input systems. In the field, inconsistent results have been reported about drivers of NUE. Some studies report good correlations between NUE and NUpE at harvest (Nyikako et al., 2014; Stahl et al., 2017), while others, focused on the reproductive phase, explain differences in NUE instead by variations in NUtE (Svečnjak and Rengel, 2006b; Stahl et al., 2015, 2019). Nonetheless, under low-N conditions, the correlation of NUpE to NUE has always been higher than that of NUtE (Berry et al., 2010; He et al., 2017), which points out the value of identifying the underlying traits of NUpE, as levers to optimize NUE under low N conditions. Our results also pointed out significant effects of GxN in the variation of NUE and its components. During the vegetative phase, this effect can reach up to 17% of the total variation observed for NUE_DM and NUpE. This highlights the occurrence of genotypic differences in plant response to N supply, which could be related to the genotype differences observed in root traits, that we highlighted as functional traits involved in NUpE variations (see below). Studies pointing out significant GxN effect on NUpE remain scarce (Nyikako et al., 2014), while GxN effect on NUtE has been more often observed (Girondé et al., 2015b; He et al., 2017; Stahl et al., 2019). In addition, GxN effects on NUE or its components have been hardly found in the field (Kessel et al., 2012; Stahl et al., 2015; Miersch et al., 2016), but have been highlighted in pots under controlled conditions (Girondé et al., 2015b; He et al., 2017). This may stem from the difficulty in discriminating N effect from other environmental variations (e.g., water status) in the field (Corlouer et al., 2019), or in quantifying N availability in the soil resulting from mineralization of organic matter. Furthermore, when observed, GxN effect on NUE was limited to harvest. In our study, the GxN effect on NUpE and NUtE was observed during vegetative growth. The interest of such dynamic studies under semi-controlled conditions is therefore the ability to identify precisely the period in the crop cycle during which GxN effects modulate the phenotype, and in particular that of the root system, with a potential effect on the performance of the crop at harvest. Thus, further investigation of the determinism of the GxN effect on NUE-related root traits would be valuable. This would require an experimental design including a larger number of genotypes and N conditions, which would imply as a prerequisite the development of high throughput phenotyping methods allowing access to dead leaves and fine roots during the whole crop cycle.

As the last main finding, we showed that the dynamic of fine-root growth was the main driver of genotypic differences in NUpE during the autumn growth under low N conditions, rather than Specific Nitrogen Uptake (SNU). Indeed, genotypes did not differ in SNU after BBCH 16–18 but significantly differed in fine-root growth for all the vegetative phase. In addition, significant correlations were found between NUpE and cumulative fine-roots biomass up to BBCH 32, but not between NUpE and SNU. This absence of genetic variability in SNU constitutes a specific characteristic of winter oilseed rape, since genotypic differences in SNU were reported for *A. thaliana* (Richard-Molard et al., 2009) and *Medicago truncatula* (Moreau et al., 2012). In our case, genotypes followed the same N acquisition course, but with different speeds. Thus, in winter oilseed rape, increasing NUE under low N conditions would rely on fine-root plasticity and/or the duration of N uptake, rather than on SNU *per se*, which highlights fine-root biomass as a promising trait for breeding N-efficient cultivars.

Many studies highlighted the crucial role of the root system, and especially fine roots, in N uptake and NUE (Hohmann et al., 2016). However, since recovering all fine roots of winter oilseed rape is impracticable in the field, their study has usually been limited to hydroponic conditions and early developmental stages (Wang et al., 2017; Qin et al., 2019). Under field or field-like conditions, root measurements have usually been limited at best to tap roots (Sieling et al., 2017). Nonetheless, we showed that the ratio of fine- to tap-root biomass differed significantly among genotypes, except at very early developmental stages, when tap roots had barely developed, thus suggesting that taproot DM cannot serve as a proxy of fine-root DM. For breeding purposes, this result clearly highlights the importance of characterizing the fine-root compartment to screen genetic resources. In our study, we considered fine-root biomass as a proxy of fine-root area, but a more detailed genotypic description of root-system architecture (e.g., length and number of lateral roots, root diameters, and branching density) could also be relevant. These traits are usually measured at early developmental stages under controlled conditions. We showed that NUE estimated at the end of inflorescence emergence was well-correlated with NUE at seed maturity. Thus, phenotyping devices that can phenotype root-system architecture accurately up to the BBCH 59 stage would be valuable for screening genetic diversity. Consequently, the next challenge for phenomics would be to extend the duration of growth supported by the existing high-throughput phenotyping platforms (Jeudy et al., 2016) up to this developmental stage.

## Data Availability Statement

In accordance with the RAPSODYN project consortium agreement, the raw data supporting the conclusions of this article will be made available by the authors upon request, at the end of the RAPSODYN project (September 2021).

## Author Contributions

AL, CB-B, CR-M, and MC were in charge of the conceptualization of the presented work. VV-C planned and executed the GR18 experiment, carried out the statistical analyses, ran the investigations, and drafted the manuscript. AL, CB-B, and CR-M designed, supervised and performed the experiments (AL and CB-B for LR15, CR-M for GR15 and GR18), were in charge of the funding acquisition, participated in the data mining, and supervised the data analysis. AL, CR-M, and VV-C were in charge of the data curation and visualization. CR-M supervised Ph.D. work of VV-C, with the contribution of MC, AL, and CB-B was in charge of the project administration. All authors reviewed, edited, and approved the submitted version of the manuscript.

## Conflict of Interest

The authors declare that the research was conducted in the absence of any commercial or financial relationships that could be construed as a potential conflict of interest.

## Footnotes

^1^https://intranet.inrae.fr/climatik_v2/ClimatikGwt.html

## Abbreviations

DM: Dry matter
GDD: Growing Degree Days
GR15: Experiment conducted in Thiverval-Grignon, France, during the 2014–2015 season
GR18: Experiment conducted in Thiverval-Grignon, France, during the 2017–2018 season
LR15: Experiment conducted in Le Rheu, France, during the 2014–2015 season
QN: Quantity of nitrogen (in a given plant compartment, in the whole plant or in the soil)
NUE_DM: Nitrogen Use Efficiency at a given sampling date, calculated as the ratio of total plant dry matter to the total amount of N available in the soil
NUE_Seed: Nitrogen Use Efficiency, calculated as the ratio of plant seed weight to the total amount of N available in the soil from sowing to harvest
NUpE: Nitrogen Uptake Efficiency
NUtE: Nitrogen Utilization Efficiency
SNU: Specific Nitrogen Uptake, calculated as the quantity of N accumulated in the plant per g of cumulative fine-root biomass.

## References

[B1] Avice J. C. Etienne P. (2014). Leaf senescence and nitrogen remobilization efficiency in oilseed rape (*Brassica napus L*.). J. Exp. Bot. 65, 3813–3824. 10.1093/jxb/eru177 24790115

[B2] Balint T. Rengel Z. (2008). Nitrogen efficiency of canola genotypes varies between vegetative stage and grain maturity. Euphytica 164, 421–432. 10.1007/s10681-008-9693-6

[B3] Bates D. M. Chambers J. M. (1992). Nonlinear models, in Statistical Models, eds Chambers S. J. M. Hastie T. J. (Pacific Grove, CA: Wadsworth and Brooks/Cole), 421–454.

[B4] Bates D. M. Watts D. G. (1988). Nonlinear Regression Analysis and Its Applications, Vol. 2. New York, NY: Wiley.

[B5] Berry P. M. Spink J. Foulkes M. J. White P. J. (2010). The physiological basis of genotypic differences in nitrogen use efficiency in oilseed rape (*Brassica napus* L.). Field Crops Res. 119, 365–373. 10.1016/j.fcr.2010.08.004

[B6] Berry P. M. Spink J. H. (2006). A physiological analysis of oilseed rape yields: past and future. J. Agric. Sci. 144, 381–392. 10.1017/S0021859606006423

[B7] Bissuel-Belaygue C. Laperche A. Bidon M. Guichard S. Leport L. Daniel L.. (2015). PERISCOPE: a new phenotyping experimental device for individual root and shoot investigations in reconStructed CanOPy until harvEst, under field-like conditions, in 14th International Rapeseed Congress (Saskatoon, SK).

[B8] Bissuel-Belaygue C. Laperche A. Guernec G. Andrianasolo F. de Oliviera A. V. Orsel M.. (2011). Leaf area index, a good functional trait for screening genetic diversity of winter oilSeed rape response to N constraint: study of a panel of 95 genotypes, in 13th International Rapeseed Congress (Prague).

[B9] Bouchet A. S. Laperche A. Bissuel-Belaygue C. Baron C. Morice J. Rousseau-Gueutin M.. (2016). Genetic basis of nitrogen use efficiency and yield stability across environments in winter rapeseed. BMC Genet. 17:131. 10.1186/s12863-016-0432-z 27628849 PMC5024496

[B10] Buckee G. K. (1994). Determination of total nitrogen in barley, malt, and beer by Kjeldahl procedures and the Dumas combustion method. J. Inst. Brew. 100, 57–64. 10.1002/jib.1994.100.2.57

[B11] Colnenne C. Meynard J. M. Roche R. Reau R. (2002). Effects of nitrogen deficiencies on autumnal growth of oilseed rape. Eur. J. Agron. 17, 11–28. 10.1016/S1161-0301(01)00140-X

[B12] Corlouer E. Gauffreteau A. Bouchet A. S. Bissuel-Bélaygue C. Nesi N. Laperche A. (2019). Envirotypes based on seed yield limiting factors allow to tackle G × E interactions. Agronomy 9:798. 10.3390/agronomy9120798

[B13] Cramer N. (1993). Umweltverträglichkeit der N-Versorgung des Rapses. Raps 11, 4–7.

[B14] Craswell E. T. Godwin D. C. (1984). The efficiency of nitrogen fertilizers applied to cereals in different climates, in Advances in Plant Nutrition, Vol. 1, eds Tinker P.B. Läuchil A. (Westport, CT: Praeger Publishers Inc.), 1–39.

[B15] Dejoux J. F. Recous S. Meynard J. M. Trinsoutrot I. Leterme P. (2000). The fate of nitrogen from winter-frozen rapeseed leaves: Mineralization, fluxes to the environment and uptake by rapeseed crop in spring. Plant Soil 218, 257–272. 10.1023/A:1014934924819

[B16] Di H. J. Cameron K. C. (2002). Nitrate leaching in temperate agroecosystems: sources, factors, and mitigation strategies. Nutr. Cycling Agroecosyst. 46, 237–256. 10.1023/A:1021471531188

[B17] Dresbøll D. B. Rasmussen I. S. Thorup-Kristensen K. (2016). The significance of litter loss and root growth on nitrogen efficiency in normal and semi-dwarf winter oilseed rape genotypes. Field Crops Res. 186, 166–178. 10.1016/j.fcr.2015.12.003

[B18] FAOSTAT (Food and Agriculture Organization of the United Nations, Statistic Division). (2017). Production Statistics. Available online at: http://faostat3.fao.org/home/E (accessed November, 2020).

[B19] Garnett T. Conn V. Kaiser B. N. (2009). Root based approaches to improving nitrogen use efficiency in plants. Plant Cell Environ. 32, 1272–1283. 10.1111/j.1365-3040.2009.02011.x 19558408

[B20] Girondé A. Etienne P. Trouverie J. Bouchereau A. Le Cahérec F. Leport L.. (2015a). The contrasting N management of two oilseed rape genotypes reveals the mechanisms of proteolysis associated with leaf N remobilization and the respective contributions of leaves and stems to N storage and remobilization during seed filling. BMC Plant Biol. 15:59. 10.1186/s12870-015-0437-1 25848818 PMC4384392

[B21] Girondé A. Poret M. Etienne P. Trouverie J. Bouchereau A. Le Cahérec F.. (2015b). A profiling approach of the natural variability of foliar N remobilization at the rosette stage gives clues to understand the limiting processes involved in the low N use efficiency of winter oilseed rape. J. Exp. Bot. 66, 2461–2473. 10.1093/jxb/erv031 25792758

[B22] He H. Yang R. Li Y. Ma A. Cao L. Wu X.. (2017). Genotypic variation in nitrogen utilization efficiency of oilseed rape (*Brassica napus* L.) under contrasting N supply in pot and field experiments. Front. Plant Sci. 8, 1–15. 10.3389/fpls.2017.01825 29163565 PMC5664426

[B23] Hohmann M. Stahl A. Rudloff J. Wittkop B. Snowdon R. J. (2016). Not a load of rubbish: simulated field trials in large-scale containers. Plant Cell Environ. 39, 2064–2073. 10.1111/pce.12737 27144906

[B24] Hotelling H. (1931). The generalization of Student's ratio. Ann. Math. Stat. 2, 360–378. 10.1214/aoms/1177732979

[B25] Jeudy C. Adrian M. Baussard C. Bernard C. Bernaud E. Bourion V.. (2016). RhizoTubes as a new tool for high throughput imaging of plant root development and architecture: test, comparison with pot grown plants and validation. Plant Methods 12, 1–18. 10.1186/s13007-016-0131-9 27279895 PMC4897935

[B26] Jullien A. Mathieu A. Allirand J. M. Pinet A. De Reffye P. Cournède P. H.. (2011). Characterization of the interactions between architecture and source sink relationships in winter oilseed rape (*Brassica napus*) using the GreenLab model. Ann. Bot. 107, 765–779. 10.1093/aob/mcq205 20980324 PMC3077979

[B27] Kant S. Bi Y. Rothstein S. J. (2011). Understanding plant response to nitrogen limitation for the improvement of crop nitrogen use efficiency. J. Exp. Bot. 62, 1499–1509. 10.1093/jxb/erq297 20926552

[B28] Kessel B. Schierholt A. Becker H. C. (2012). Nitrogen use efficiency in a genetically diverse set of winter oilseed rape. Crop Sci. 52, 2546–2554. 10.2135/cropsci2012.02.0134

[B29] Kjeldahl J. (1883). A new method for the determination of nitrogen in organic matter. Z. Anal. Chem. 22, 366–382. 10.1007/BF01338151

[B30] Koscielny C. B. Gulden R. H. (2012). Seedling root length in *Brassica napus* L. is indicative of seed yield. Can. J. Plant Sci. 92, 1229–1237. 10.4141/cjps2012-070

[B31] Lancashire P. D. Bleiholder H. Langelüddecke P. Strauss R. Vanden Boom T. Weber E.. (1991). An uniform decimal code for growth stages of crops and weeds. Ann. Appl. Biol. 119, 561–570. 10.1111/j.1744-7348.1991.tb04895.x

[B32] Laperche A. Devienne-Barret F. Maury O. Le Gouis J. Ney B. (2006). A simplified conceptual model of carbon/nitrogen functioning for QTL analysis of winter wheat adaptation to nitrogen deficiency. Theor. Appl. Genet. 113, 1131–1146. 10.1007/s00122-006-0373-4 16909280

[B33] Lasdon L. Fox R. L. Ratne M. W. (1974). Nonlinear optimization using the generalized reduced gradient method. Fr. J. Autom. Comput. Sci. Oper. Res. 8, 73–104. 10.1051/ro/197408V300731

[B34] Lemaire G. Gastal F. (1997). N uptake and distribution in plant canopies, in Diagnosis of the Nitrogen Status in Crops, ed Lemaire G. (Berlin: Springer), 3–43.

[B35] Lemaire G. van Oosterom E. Jeuffroy M. H. Gastal F. Massignam A. (2008). Crop species present different qualitative types of response to N deficiency during their vegetative growth. Field Crop Res. 105, 253–265. 10.1016/j.fcr.2007.10.009

[B36] Louvieaux J. Spanoghe M. Hermans C. (2020). Root morphological traits of seedlings are predictors of seed yield and quality in winter oilseed rape hybrid cultivars. Front. Plant Sci. 11:568009. 10.3389/fpls.2020.568009 33178235 PMC7593254

[B37] Malagoli P. Lainé P. Rossato L. Ourry A. (2005a). Dynamics of nitrogen uptake and mobilization in field-grown winter oilseed rape (*Brassica napus*) from stem extension to harvest: I. Global N flows between vegetative and reproductive tissues in relation to leaf fall and their residual N. Ann. Bot. 95, 853–861. 10.1093/aob/mci091 15701662 PMC4246740

[B38] Malagoli P. Lainé P. Rossato L. Ourry A. (2005b). Dynamics of nitrogen uptake and mobilization in field-grown winter oilseed rape (*Brassica napus*) from stem extension to harvest: II. An ^15^N-labelling-based simulation model of N partitioning between vegetative and reproductive tissues. Ann. Bot. 95, 1187–1198. 10.1093/aob/mci131 15802311 PMC4246903

[B39] Miersch S. Gertz A. Breuer F. Schierholt A. Becker H. C. (2016). Influence of the semi-dwarf growth type on nitrogen use efficiency in winter oilseed rape. Crop Sci. 56, 2952–2996. 10.2135/cropsci2016.01.0044

[B40] Moll R. H. Kamprath E. J. Jackson W. (1982). Analysis and interpretation of factors which contribute to efficiency of nitrogen utilization. Agron. J. 74, 562–564. 10.2134/agronj1982.00021962007400030037x

[B41] Moreau D. Burstin J. Aubert G. Huguet T. Ben C. Prosperi J. M.. (2012). Using a physiological framework for improving the detection of quantitative trait loci related to nitrogen nutrition in *Medicago truncatula*. Theor. Appl. Genet. 124, 755–768. 10.1007/s00122-011-1744-z 22113590

[B42] Nyikako J. Schierholt A. Kessel B. Becker H. C. (2014). Genetic variation in nitrogen uptake and utilization efficiency in a segregating DH population of oilseed rape. Euphytica 199, 3–11. 10.1007/s10681-014-1201-6

[B43] Qin L. Walk T. C. Han P. Chen L. Zhang S. Li Y.. (2019). Adaptation of roots to nitrogen deficiency revealed by 3D quantification and proteomic analysis. Plant Physiol. 179, 329–347. 10.1104/pp.18.00716 30455286 PMC6324228

[B44] R Core Team (2018). R: A Language and Environment for Statistical Computing. Vienna: R Foundation for Statistical Computing. Available online at: http://www.R-project.org/

[B45] Rakotoson T. Dusserre J. Letourmy P. Ramonta I. R. Caod T. V. Ramanantsoanirina A.. (2017). Genetic variability of nitrogen use efficiency in rainfed upland rice. Field Crop Res. 213, 194–203. 10.1016/j.fcr.2017.07.023

[B46] Raun W. R. Johnson G. V. (1999). Improving nitrogen use efficiency for cereal production. Agron. J. 91, 357–363. 10.2134/agronj1999.00021962009100030001x

[B47] Richard-Molard C. Brun F. Chelle M. Ney B. (2009). Modelling N nutrition impact on plant functioning and root architecture in various genotypes of *Arabidopsis thaliana*. Comp. Biochem. Physiol. Part A Mol. Integr. Physiol. 153:S229. 10.1016/j.cbpa.2009.04.634

[B48] Richard-Molard C. Krapp A. Brun F. Ney B. Daniel-Vedele F. Chaillou S. (2008). Plant response to nitrate starvation is determined by N storage capacity matched by nitrate uptake capacity in two Arabidopsis genotypes. J. Exp. Bot. 59, 779–791. 10.1093/jxb/erm363 18304979

[B49] Robinson D. (2004). Scaling the depths: below-ground allocation in plants, forests, and biomes. Funct. Ecol. 18, 290–295. 10.1111/j.0269-8463.2004.00849.x

[B50] Rossato L. Lainé P. Ourry A. (2001). Nitrogen storage and remobilization in *Brassica napus* L. during the growth cycle: nitrogen fluxes within the plant and changes in soluble protein patterns. J. Exp. Bot. 52, 1655–1663. 10.1093/jexbot/52.361.1655 11479330

[B51] Rothstein S. J. (2007). Returning to our roots: making plant biology research relevant to future challenges in agriculture. Plant Cell 19, 2695–2699. 10.1105/tpc.107.053074 17873097 PMC2048712

[B52] Sainju U. M. Stevens W. B. Caesar-TonThat T. Liebig M. A. (2012). Soil greenhouse gas emissions affected by irrigation, tillage, crop rotation, and nitrogen fertilization. J. Environ. Qual. 41, 1774–1786. 10.2134/jeq2012.0176 23128735

[B53] Schulte auf'm Erley G. Behrens T. Ulas A. Wiesler F. Horst W. J. (2011). Agronomic traits contributing to nitrogen efficiency of winter oilseed rape cultivars. Field Crops Res. 124, 114–123. 10.1016/j.fcr.2011.06.009

[B54] Sieling K. Böttcher U. Kage H. (2017). Sowing date and N application effects on tap root and above ground dry matter of oilseed rape in autumn. Eur. J. Agron. 83, 40–46. 10.1016/j.eja.2016.11.006

[B55] Stahl A. Friedt W. Wittkop B. Snowdon R. J. (2015). Complementary diversity for nitrogen uptake and utilisation efficiency reveals broad potential for increased sustainability of oilseed rape production. Plant Soil 400, 245–262. 10.1007/s11104-015-2726-8

[B56] Stahl A. Pfeifer M. Frisch M. Wittkop B. Snowdon R. J. (2017). Recent genetic gains in nitrogen use efficiency in oilseed rape. Front. Plant Sci. 8:963. 10.3389/fpls.2017.00963 28638399 PMC5461335

[B57] Stahl A. Vollrath P. Samans B. Frisch M. Wittkop B. Snowdon R. J. (2019). Effect of breeding on nitrogen use efficiency-associated traits in oilseed rape. J. Exp. Bot. 70, 1969–1986. 10.1093/jxb/erz044 30753580 PMC6436158

[B58] Svečnjak Z. Rengel Z. (2006a). Canola cultivars differ in nitrogen utilization efficiency at vegetative stage. Field Crops Res 97, 221–226. 10.1016/j.fcr.2005.10.001

[B59] Svečnjak Z. Rengel Z. (2006b). Nitrogen utilization efficiency in canola cultivars at grain harvest. Plant Soil 283, 299–307. 10.1007/s11104-006-0020-5

[B60] Thomas C. L. Graham N. S. Hayden R. Meacham M. C. Neugebauer K. Nightingale M.. (2016). High-throughput phenotyping (HTP) identifies seedling root traits linked to variation in seed yield and nutrient capture in field-grown oilseed rape (*Brassica napus* L.). Ann. Bot. 118, 655–665. 10.1093/aob/mcw046 27052342 PMC5055618

[B61] Ulas A. Schulte Auf'm Erley G. Kamh M. Wiesler F. Horst W. J. (2012). Root-growth characteristics contributing to genotypic variation in nitrogen efficiency of oilseed rape. J. Plant Nutr. Soil Sci. 175, 489–498. 10.1002/jpln.201100301

[B62] Wang J. Dun X. Shi J. Wang X. Liu G. Wang H. (2017). Genetic dissection of root morphological traits related to nitrogen use efficiency in *Brassica napus* L. under two contrasting nitrogen conditions. Front. Plant Sci. 8, 1–15. 10.3389/fpls.2017.01709 29033971 PMC5626847

[B63] Yuan P. Ding G. D. Cai H. M. Jin K. M. Broadley M. R. Xu F. S.. (2016). A novel *Brassica*-rhizotron system to unravel the dynamic changes in root system architecture of oilseed rape under phosphorus deficiency. Ann. Bot. 118, 173–184. 10.1093/aob/mcw083 27279575 PMC4970355

