# Supplementary material for: Nitrogen Uptake Efficiency, Mediated by Fine Root Growth, Early Determines Temporal and Genotypic Variations in Nitrogen Use Efficiency of Winter Oilseed Rape
Source: Front Plant Sci. 2021 May 13;12:641459. doi: 10.3389/fpls.2021.641459 (PMC8155714; doi:10.3389/fpls.2021.641459)
Supplement: Supplementary file 1 [file Data_Sheet_1.PDF]

Supplementary Material

Table S1. Winter oilseed rape genotypes investigated, by experiment, release year and type of variety. Experiment codes: LR15, Le Rheu 2014-2015; GR15, Grignon 2014-2015; GR18, Grignon 2017-2018. Type codes: ++', high glucosinolate and erucic acid contents; 00', low glucosinolate and erucic acid contents.

Genotype	Experiment	Year of release	Type
AMBER	LR15, GR18	2003	00'
ASTRID	LR15	2004	00'
AVISO	LR15, GR15, GR18	2000	00'
EXPRESS	LR15, GR18	1993	00'
MOHICAN	LR15, GR18	1995	00'
MONTEGO	LR15	2001	00'
OLESKI	GR18	<1980	++'

Table S2. Number of plants and replicates sampled in each experiment under each N condition. The upper part of the table presents the number of samples collected per experiment (by grouping all genotype replicates and sampling dates) or per sampling date (by grouping all genotype replicates) under each N condition and each experiment. The lower part presents the number of replicates collected at each sampling date for each genotype, N condition and experiment. Four samplings were carried out in the LR15 experiment. Five samplings were carried out in the GR15 and GR18 experiments. The same pattern of replicates was applied at each sampling date.

	LR15		GR15		GR18	
	Low-N	High-N	Low-N	High-N	Low-N	High-N
**Number of plants in the whole experiment**	120	120	30	30	185	30
**Number of plants per sampling date**	30	30	6	6	37	6
**Number of replicates per sampling date for each genotype**						
AVISO	5	5	6	6	7	6
AMBER	5	5	-	-	7	-
ASTRID	5	5	-	-	-	-
EXPRESS	5	5	-	-	7	-
MOHICAN	5	5	-	-	8	-
MONTEGO	5	5	-	-	-	-
OLESKI	-	-	-	-	8	-

Table S3. Analysis of the regressions between NUE_Seed measured at seed maturity (BBCH 84-89) (y) and NUE_DM measured at each sampling date (x), within each separate experiment and N condition. Regressions were carried out considering genotype mean values either in each N condition (LN: Low N-condition, HN: high N-condition, n = 5 in LR15 and n = 7 to 8 in GR18 at each sampling date) or by pooling both N conditions (LN + HN, n = 10 in LR15 and n = 7 to 13 in GR18 at each sampling date). Analyses on GR15 and GR18 HN were trivial as only one value was available (AVISO). Significance code: *** P-value < 0.001, ** P-value < 0.01, * P-value < 0.05, ns: non-significant. The non-significant relationship obtained in LR15 under the high N condition at BBCH 84-89 was shown to be significant when using all individual plant samples (n = 30, $R^2 = 0.7516$ ***, data not shown).

Growing Stage	Experiment	N condition	Equation	R^2	*P*-value	Significance level
**BBCH 84-89**	GR18	LN + HN	y = 0.2685x - 0.731	0.86	0.0073	**
	GR18	LN	y = 0.317x - 3.82	0.88	0.0188	*
	LR15	LN + HN	y = 0.208x +2.061	0.79	0.0001	***
	LR15	LN	y = 0.236x + 0.094	0.47	0.0132	*
	LR15	HN	y = 0.167x + 3.818	0.29	0.2730	ns
**BBCH 68-71**	LR15	LN + HN	y = 0.279x - 1.05	0.87	1.4E-06	***
	LR15	LN	y = 0.364x - 6.16	0.76	0.0230	*
	LR15	HN	y = 0.180x + 2.96	0.53	0.0224	*
**BBCH 59**	GR18	LN + HN	y = 0.762x - 13.2	0.71	0.0090	**
	GR18	LN	y = 0.586x - 6.02	0.66	0.0480	*
**BBCH 30-32**	GR18	LN + HN	y = 0.588x + 1	0.57	0.0309	*
	GR18	LN	y = 0.617x + 0.114	0.05	0.7297	ns
	LR15	LN + HN	y = 0.580x + 7.2	0.69	0.0017	**
	LR15	LN	y = 0.329x + 11	0.24	0.3979	ns
	LR15	HN	y = 0.599x + 6.7	0.58	0.0800	ns
**BBCH 19**	GR18	LN + HN	y = 0.285x + 10.4	0.08	0.4920	ns
	GR18	LN	y = 0.154x + 13.4	0.03	0.7328	ns
**BBCH16-18**	GR18	LN + HN	y = 0.154x - 14.3	0.00	0.9743	ns
	GR18	LN	y = -5.24x + 22	0.24	0.4060	ns
	LR15	LN + HN	y = 2.32x + 9.71	0.20	0.1418	ns
	LR15	LN	y = -1.87x + 20.1	0.17	0.4185	ns
	LR15	HN	y = 2.62x + 7.74	0.59	0.0753	ns

Table S4. Main variables depicting N use efficiency and root system growth of five winter oilseed rape genotypes grown under the low-N condition during the crop cycle (GR18 experiment). Five key phenological stages were targeted: BBCH 16 (beginning of rosette growth), BBCH 19 (mid rosette development), BBCH 32 (beginning of stem elongation), BBCH 59 (just before flower opening), and BBCH 89 (seed maturity). Data were expressed as the mean of each variable for each genotype at a given sampling date (n = 7 to 8 at each sampling date). Means with the same letter for a given sampling date do not differ significantly according to Tukey's test (*P*-value ≤ 0.05). *na*: not available.

Trait / Stage	Unit	Genotype									
		AMBER		AVISO		EXPRESS		MOHICAN		OLESKI	
**Whole plant N-quantity**	gN plant^{-1}										
BBCH 16		0.02	a	0.02	a	0.01	a	0.02	a	0.03	b
BBCH 19		0.13	bc	0.12	b	0.08	a	0.12	b	0.16	c
BBCH 32		0.24	a	0.25	a	0.20	a	0.23	a	0.24	a
BBCH 59		0.29	a	0.30	a	0.26	a	0.29	a	0.28	a
BBCH 89		0.32	ab	0.36	ab	0.29	ab	0.40	b	0.30	a
**N Uptake Efficiency (NUpE)**	gN gN^{-1}										
BBCH 16		0.06	a	0.07	a	0.05	a	0.07	a	0.09	b
BBCH 19		0.41	bc	0.37	b	0.26	a	0.37	b	0.51	c
BBCH 32		0.68	a	0.71	a	0.66	a	0.67	a	0.69	a
BBCH 59		0.76	a	0.78	a	0.68	a	0.75	a	0.74	a
BBCH 89		0.65	ab	0.74	ab	0.61	ab	0.83	b	0.61	a
**N Utilization Efficiency (NUtE)**	g gN^{-1}										
BBCH 16		19.8	a	19.8	a	19.7	a	20.0	a	20.6	a
BBCH 19		31.6	b	29.4	ab	26.8	a	28.8	ab	28.1	ab
BBCH 32		37.9	c	34.1	ab	35.5	b	34.2	ab	32.6	a
BBCH 59		47.3	a	54.1	b	43.9	a	55.2	b	42.1	a
BBCH 89		92.8	b	86.7	b	80.3	ab	86.9	b	76.2	a
**Specific N-uptake**	g gN^{-1}										
BBCH 16		0.0026	b	0.0027	b	0.0015	a	0.0014	a	0.0009	a
BBCH 19		0.0014	a	0.0013	a	0.0012	a	0.0012	a	0.0014	a
BBCH 32		0.0006	a	0.0006	a	0.0006	a	0.0006	a	0.0006	a
BBCH 59		0.0003	a	0.0003	a	0.0003	a	0.0003	a	0.0004	a
BBCH 89		*na*		*na*		*na*		*na*		*na*	
**Total root dry matter**	g plant^{-1}										
BBCH 16		0.09	ab	0.10	ab	0.08	a	0.12	b	0.18	c
BBCH 19		1.16	b	0.96	ab	0.62	a	1.03	b	1.29	b
BBCH 32		2.78	a	2.44	a	2.67	a	2.22	a	2.48	a
BBCH 59		3.57	ab	3.93	ab	3.31	a	4.29	b	3.25	a
BBCH 89		3.18	b	3.29	b	1.68	a	2.87	b	1.93	a
**Taproot dry matter**	g plant^{-1}										
BBCH 16		0.02	bc	0.02	b	0.01	a	0.03	bc	0.03	c
BBCH 19		0.61	cd	0.39	ab	0.26	a	0.54	bc	0.82	d
BBCH 32		1.64	a	1.37	a	1.56	a	1.31	a	1.75	a
BBCH 59		2.21	ab	2.54	b	1.68	a	2.67	b	2.18	ab
BBCH 89		2.20	b	2.48	b	1.07	a	2.26	b	1.25	a
**Fine root dry matter**	g plant^{-1}										
BBCH 16		0.07	a	0.08	a	0.06	a	0.10	a	0.15	b
BBCH 19		0.55	ab	0.57	b	0.36	a	0.48	ab	0.47	ab
BBCH 32		1.14	b	1.07	b	1.11	b	0.90	ab	0.73	a
BBCH 59		1.36	ab	1.38	ab	1.63	b	1.62	b	1.08	a
BBCH 89		0.97	a	0.81	a	0.61	a	0.62	a	0.68	a
**Fine root / Total root dry matter**	g plant^{-1}										
BBCH 16		74.0	a	77.1	ab	81.9	b	78.9	ab	81.3	b
BBCH 19		47.6	b	59.4	c	58.3	c	47.7	b	36.6	a
BBCH 32		41.2	b	44.2	b	41.8	b	41.2	b	29.5	a
BBCH 59		40.1	ab	35.2	a	49.3	b	37.7	a	33.3	a
BBCH 89		30.0	a	25.6	a	36.4	a	22.4	a	35.2	a

Table S5. Main variables depicting N use efficiency and root system growth of five winter oilseed rape genotypes grown under the low-N condition during the crop cycle (LR15 experiment). Four key phenological stages were targeted: BBCH 18 (beginning of rosette growth), BBCH 31 (beginning of stem elongation), BBCH 68 (end of the flowering period), and BBCH 84 (seed maturity). Data were expressed as the mean of each variable for each genotype at a given sampling date (n = 6 at each sampling date). Means with the same letter for a given sampling date do not differ significantly according to Tukey's test (P-value ≤ 0.05). *na*: not available.

Trait / Stage	Unit	Genotype					
		AMBER	ASTRID	AVISO	EXPRESS	MOHICAN	MONTEGO
**Whole plant N-quantity**	gN plant^{-1}						
BBCH 18		0.01 a	0.02 a	0.03 b	0.02 ab	0.02 ab	0.02 ab
BBCH 31		0.08 bc	0.07 c	0.13 a	0.08 bc	0.11 ab	0.12 ab
BBCH 68		0.22 a	0.31 a	0.29 a	0.31 a	0.28 a	0.26 a
BBCH 84		0.32 a	0.38 a	0.34 a	0.31 a	0.37 a	0.35 a
**N Uptake Efficiency (NUpE)**	gN gN^{-1}						
BBCH 18		0.05 a	0.06 a	0.12 b	0.09 ab	0.08 ab	0.10 ab
BBCH 31		0.26 bc	0.22 c	0.44 a	0.27 bc	0.37 ab	0.39 ab
BBCH 68		0.59 a	0.83 a	0.79 a	0.82 a	0.76 a	0.70 a
BBCH 84		0.79 a	0.94 a	0.85 a	0.75 a	0.92 a	0.87 a
**N Utilization Efficiency (NUtE)**	g gN^{-1}						
BBCH 18		24.04 abc	23.32 bc	25.67 ab	26.32 a	22.93 c	24.69 abc
BBCH 31		34.43 a	34.01 a	38.72 ab	35.23 a	38.94 ab	41.87 b
BBCH 68		94.01 a	86.08 a	82.45 a	63.07 b	86.69 a	84.22 a
BBCH 84		87.33 a	73.41 b	82.91 a	70.32 b	86.58 a	77.90 ab
**Total root dry matter**	g plant^{-1}						
BBCH 18		0.07 a	0.09 a	0.15 b	0.14 ab	0.11 ab	0.12 ab
BBCH 31		0.96 bc	0.91 c	1.91 a	1.24 abc	1.71 ab	1.76 a
BBCH 68		4.34 a	4.55 a	4.59 a	3.24 a	5.50 a	4.36 a
BBCH 84		4.47 a	3.81 ab	5.23 a	2.56 b	4.09 ab	4.66 a
**Taproot dry matter**	g plant^{-1}						
BBCH 18		0.03 a	0.04 a	0.08 b	0.06 ab	0.05 ab	0.06 ab
BBCH 31		0.50 bc	0.40 c	1.02 a	0.53 bc	0.91 ab	0.86 abc
BBCH 68		3.12 a	3.07 a	3.24 a	2.53 a	3.19 a	2.92 a
BBCH 84		3.54 ab	2.52 bc	3.67 a	1.58 c	3.11 ab	3.20 ab
**Fine root dry matter**	g plant^{-1}						
BBCH 18		0.03 b	0.05 ab	0.07 a	0.08 a	0.06 ab	0.07 ab
BBCH 31		0.46 b	0.51 ab	0.90 a	0.72 ab	0.81 ab	0.90 a
BBCH 68		1.38 a	1.48 a	1.35 a	1.16 a	1.45 a	1.44 a
BBCH 84		0.93 a	1.07 ab	1.54 b	0.98 ab	1.01 ab	1.46 ab
**Fine root / Total root dry matter**	g plant^{-1}						
BBCH 18		51.2 a	57.4 a	48.4 a	58.3 a	53.8 a	53.4 a
BBCH 31		48.8 a	56.0 a	47.3 a	57.7 a	47.8 a	51.2 a
BBCH 68		28.6 a	35.1 a	29.4 a	40.2 a	31.9 a	33.2 a
BBCH 84		20.7 c	28.3 abc	29.3 abc	38.3 a	22.1 bc	31.1 ab

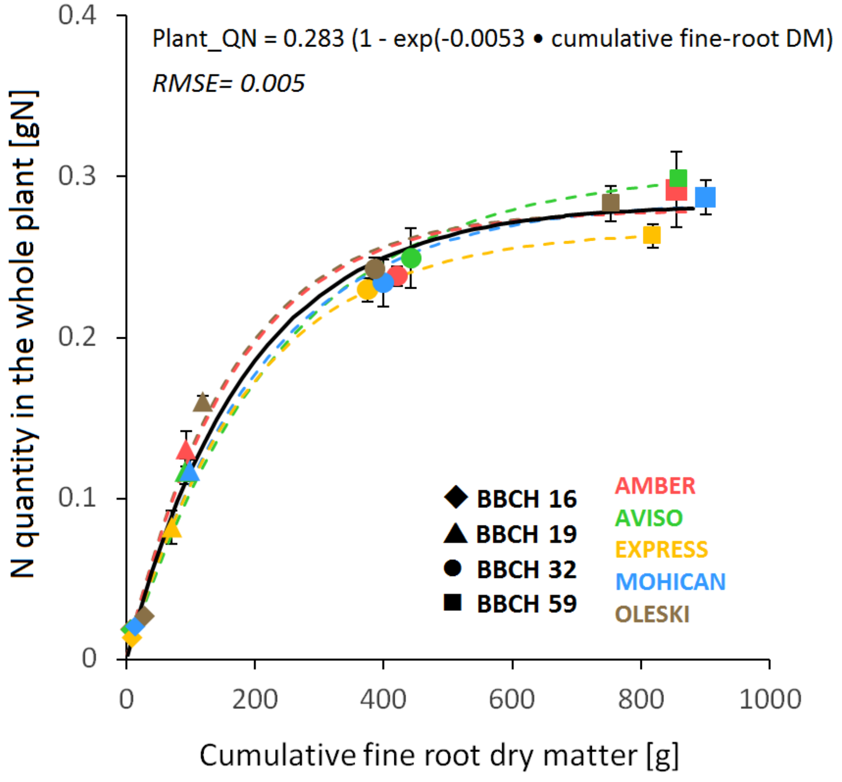

Figure S1. Relationship between the total amount of N in the plant and the cumulative fine-root biomass for five winter oilseed rape genotypes grown under the low-N condition during the vegetative growth (GR18 experiment). Colored symbols indicate the mean value per genotype for the measured amount of N in the whole plant and the estimated amount of cumulative fine-root dry matter. Four key phenological stages were targeted: BBCH 16 (beginning of rosette growth, diamonds), BBCH 19 (mid rosette development, triangles), BBCH 32 (beginning of stem elongation, circles) and BBCH 59 (just before flower opening, squares). Error bars depict standard errors of the mean of each genotype (n = 7 to 8 at each phenological stage). The best equation to fit the relationship was shown to be a negative exponential function ($y = a\ (1 - exp^{-bx})$, with y = N quantity in the whole plant and x = cumulative fine-root dry matter), either for each genotype (dashed lines) or for the mean of all genotypes (solid black line). RMSE indicates the quality of the model. Parameters were a = 0.283 and b = 0.0053 for the mean curve and showed no significant differences between genotypes (*P*-value = 0.17 and 0.96 for a and b respectively).
